# Protein kinase C inhibitor Gö6976 but not Gö6983 induces the reversion of E- to N-cadherin switch and metastatic phenotype in melanoma: identification of the role of protein kinase D1

**DOI:** 10.1186/s12885-016-3007-5

**Published:** 2017-01-05

**Authors:** Messaouda Merzoug-Larabi, Caroline Spasojevic, Marianne Eymard, Caroline Hugonin, Christian Auclair, Manale Karam

**Affiliations:** 1 LBPA, ENS Cachan, CNRS, Université Paris-Saclay, Cachan, 94235 France; 2 Département de Génétique, Institut Curie, Unité de Pharmacogénomique, Paris, 75248 France; 3 Qatar Biomedical Research Institute, Hamad Bin Khalifa University, Qatar Foundation, Doha, 5825 Qatar

**Keywords:** Gö6976, Protein kinase C, Protein kinase D1, Cadherin switch, Melanoma, Metastasis

## Abstract

**Background:**

Melanoma is a highly metastatic type of cancer that is resistant to all standard anticancer therapies and thus has a poor prognosis. Therefore, metastatic melanoma represents a significant clinical problem and requires novel and effective targeted therapies. The protein kinase C (PKC) family comprises multiple isoforms of serine/threonine kinases that possess distinct roles in cancer development and progression. In this study, we determined whether inhibition of PKC could revert a major process required for melanoma progression and metastasis; i.e. the E- to N-cadherin switch.

**Methods:**

The cadherin switch was analyzed in different patient-derived primary tumors and their respective metastatic melanoma cells to determine the appropriate cellular model (aggressive E-cadherin-negative/N-cadherin-positive metastasis-derived melanoma cells). Next, PKC inhibition in two selected metastatic melanoma cell lines, was performed by using either pharmacological inhibitors (Gö6976 and Gö6983) or stable lentiviral shRNA transduction. The expression of E-cadherin and N-cadherin was determined by western blot. The consequences of cadherin switch reversion were analyzed: cell morphology, intercellular interactions, and β-catenin subcellular localization were analyzed by immunofluorescence labeling and confocal microscopy; cyclin D1 expression was analyzed by western blot; cell metastatic potential was determined by anchorage-independent growth assay using methylcellulose as semi-solid medium and cell migration potential by wound healing and transwell assays.

**Results:**

Gö6976 but not Gö6983 reversed the E- to N-cadherin switch and as a consequence induced intercellular interactions, profound morphological changes from elongated mesenchymal-like to cuboidal epithelial-like shape, β-catenin translocation from the nucleus to the plasma membrane inhibiting its oncogenic function, and reverting the metastatic potential of the aggressive melanoma cells. Comparison of the target spectrum of these inhibitors indicated that these observations were not the consequence of the inhibition of conventional PKCs (cPKCs), but allowed the identification of a novel serine/threonine kinase, i.e. protein kinase Cμ, also known as protein kinase D1 (PKD1), whose specific inhibition allows the reversion of the metastatic phenotype in aggressive melanoma.

**Conclusion:**

In conclusion, our study suggests, for the first time, that while cPKCs don’t embody a pertinent therapeutic target, inhibition of PKD1 represents a novel attractive approach for the treatment of metastatic melanoma.

**Electronic supplementary material:**

The online version of this article (doi:10.1186/s12885-016-3007-5) contains supplementary material, which is available to authorized users.

## Background

Melanoma is a highly metastatic and deadly type of cancer that arises from melanocytes, melanin-producing cells residing in the basal layer of the epidermis and necessary for protection of skin cells from deleterious effects of ultraviolet light. The incidence of melanoma is increasing very fast worldwide [1]. When diagnosed early, most patients with primary melanoma can be cured by surgical resection. However, if not detected and removed early, melanoma cells can metastasize rapidly. Metastatic melanoma has historically been considered an untreatable disease, where standard treatment options produced modest response rates and failure to improve overall survival [2, 3]. Recently, the treatment landscape for advanced melanoma was revolutionized by the development of new targeted and immune therapeutic strategies. Particularly, BRAF/MAPK pathway inhibitors and immune checkpoint inhibitors have proven to significantly improve survival in melanoma patients in comparison to traditional therapeutics [4, 5]. However, many patients develop resistance to MAPK inhibitor therapies and most patients do not respond to immunotherapies. Therefore, metastatic melanoma represents an important health problem and requires novel and effective targeted therapies.

In human epidermis, normal melanocytes interact with keratinocytes through the adhesion molecule E-cadherin. This communication maintains differentiation state of melanocytes and control their proliferation and migration [6, 7]. Transformation of melanocytes into melanoma entails a number of genetic and environmental factors involving cell adhesion and growth regulatory genes. One key event allowing melanoma progression is the loss of E-cadherin and gain of another member of classical cadherins, i.e. N-cadherin [8, 9]. This cadherin switch results in the loss of keratinocyte-mediated growth and motility control [6] and enables melanoma cells to interact directly with N-cadherin-expressing stromal cells from the dermis, such as fibroblasts and vascular or lymphoid endothelial cells [10]. These events are crucial to allow melanoma cells to metastasize.

E- and N-cadherin are members of the classical cadherin family that play an important role in cell-cell adhesion regulating morphogenesis during embryonic development and maintaining integrity in developed tissues [11]. These transmembrane glycoproteins mediate calcium-dependent intercellular adhesion in a homophilic manner. Cadherin-mediated cell-cell junctions are formed as a result of interaction between extracellular domains of identical cadherins, which are located on the membrane of neighboring cells. The stability of these adhesive junctions is insured by binding of the intracellular cadherin domain with the actin cytoskeleton through the cytoplasmic proteins α-, β- and γ-catenins [12]. The E-cadherin is expressed by most normal epithelial tissues and N-cadherin is typically expressed by mesenchymal cells which, in contrast to epithelial cells, are non-polarized, elongated, less adherent between each other, motile and resistant to anoikis [13]. However, many epithelium-derived cancer cells have lost E-cadherin expression and inappropriately express N-cadherin. This cadherin switch has been shown to promote tumor growth, motility and invasion through a process called epithelial-mesenchymal transition (EMT) [6, 14–16] and to be associated with metastasis and poor prognosis in patients [17, 18]. Since functional restoration of E-cadherin or depletion of N-cadherin in melanoma cells inhibits tumor cell growth, motility and invasion in vitro and reduces tumorigenicity in vivo [6, 19], identification of molecular mechanisms reverting the E- to N-cadherin switch may be a way to identification of new potential therapeutic targets for melanoma treatment.

The protein kinase C (PKC) family of serine/threonine kinases includes multiple isozymes that are divided into three groups, conventional, novel, or atypical, depending on their requirements for Ca^2+^ or diacylglycerol for activation [20]. Signaling through PKCs is induced by a remarkable number of stimuli, including G-protein-coupled receptor agonists and growth factors. Individual PKC isozymes regulate a varied array of biological processes including cell proliferation, survival, migration and apoptosis. They are involved in the development and progression of different types of cancer including melanoma [21, 22]. PKCu, also known as PKD1 (protein kinase D1) was initially described as a member of the PKC family [23]. However, PKD1 possesses distinct substrate specificity [24] and has therefore recently been classified as a novel subgroup of the calcium/calmodulin-dependent kinase (CAMK) family [25]. Despite the new classification of PKD1, this serine/threonine kinase shares with conventional and novel PKCs common activators, such as phorbol esters [24], as well as potent inhibitors such as Gö6976 [26, 27]. Phorbol esters and Gö6976 inhibitor has been shown to regulate cell junctions, cadherin expression and migration [28, 29]. However, to date, the role of PKCs in cadherin switch and melanoma progression remains unknown.

In the present study, we analyzed whether PKC inhibitors Gö6976 and Gö6983 would revert the E- to N- cadherin switch and metastatic phenotype in aggressive melanoma cells.

## Methods

### Antibodies and materials

The primary antibodies used were rabbit anti-E-cadherin, rabbit anti-N-cadherin, mouse anti-β-catenin and rabbit anti-PKD1 (1/500 for western blot and 1/50 for immunofluorescence; Santa Cruz Biotechnology, Santa Cruz, CA), goat anti-actin (1/200 for western blot; Santa Cruz Biotechnology, Santa Cruz, CA) and rabbit anti-cyclin D1 (1/1000 for western blot, Cell signaling technology, Denvers, MA). Horseradish peroxidase-conjugated secondary antibodies used were goat anti-rabbit IgG (1/2000; Dako, Glostrup, Denmark), rabbit anti-goat IgG (1/2000 Santa Cruz Biotechnology) and goat anti-mouse IgG (1/5000; Rockland, Gilbertsville, PA). The Alexa Fluor-conjugated secondary antibodies were Alexa-Fluor-594-conjugated donkey anti-rabbit IgG, Alexa-Fluor-488-conjugated donkey anti-mouse IgG conjugated (1/200; Invitrogen, Cergy-Pontoise, France). Actin was stained with Alexa-Fluor-488-conjugated phalloidin (1/50; Invitrogen, Cergy-Pontoise, France). The nucleus was stained with 4',6-diamidino-2-phenylindole DAPI (1/50000; Invitrogen, Cergy-Pontoise, France). Gö6976 and Gö6983 were purchased from Calbiochem (Darmstadt, Germany). All other biochemicals were from Sigma-Aldrich (St. Louis, MO).

### Cell culture

Primary (T1 and I5), their respective lymph-node metastasis (G1 and M2), and the cutaneous metastasis (M4T2) melanoma cell lines were obtained from 70-77-year old patients from Institut Gustave Roussy (Villejuif, France), as previously described [30]. These cells were cultured in RPMI medium supplemented with 10% fetal bovine serum (FBS), 100 units/mL penicillin and 100 μg/mL streptomycin (P/S), and 1 mM sodium pyruvate (complete medium).

### Stable shRNA lentiviral transduction

For stable PKD1 depletion, cells were infected with human PKCμ shRNA (sc-36245-v) or control shRNA (sc-108080) lentiviral transduction particles from Santa Cruz Biotechnology (Santa Cruz, CA) according to the manufacturer’s protocol. Stable clones expressing the shRNA were selected with 2 μg/mL puromycin.

### Western blot analysis

Cells were lysed for 20 min at 4 °C in 50 mM Tris–HCl pH 7.4, 150 mM NaCl, 1 mM EDTA, 100 mM sodium fluoride, 10 mM tetra-sodium diphosphate decahydrate, 2 mM sodium orthovanadate, 1 mM phenylmethylsulfonylfluoride, 10 μg/mL aprotinin and 1% Nonidet P-40. Lysates were clarified by centrifugation at 14,000 rpm for 10 min at 4 °C. 30–80 μg of total protein extracts were separated by SDS-PAGE and transferred onto nitrocellulose membranes. These were incubated with the specific antibodies overnight at 4 °C and revealed by enhanced chemiluminescence (Amersham, GE Healthcare, UK).

### MTT assay

Cells were seeded in quadruplicates into 96-well plates at a density of 1,000 cells per well in complete medium and incubated for 1 to 6 days at 37 °C, 5% CO_2_. On the day of analysis, cells were incubated with 0.5 mg/ml MTT for 2 h at 37 °C. Then, 10% SDS were added to each well and incubated for 16 h at 37 °C. Absorbance value (OD) was measured at 570 nm.

### Colony formation assay

Cells were resuspended in 2.5 mL of methylcellulose (0.8%) prepared in complete medium containing vehicle control (DMSO), 1 μM Gö6976 or 1 μM Gö6983. Cells were then plated in uncoated 35 mm culture dishes and incubated at 37 °C in a humidified atmosphere at 5% CO2 for 3 weeks. Colonies were then photographed and counted under a light microscope using a grid.

### Wound healing assay

The scratch wound was created using 200 μL sterile pipette tip in a confluent cell culture pre-treated for 24 h with DMSO, 1 μM Gö6976 or 1 μM Gö6983. The scratch area was washed and cells were re-incubated with the same inhibitors. The images were taken at 0, 16 and 24 h. The lines show the area where the scratch wound was created.

### Transwell migration assay

24-well transwell chambers (Corning Costar, Corning, NY, USA) with 8.0-μm pore size polycarbonate membrane were used. 100,000 cells were plated in duplicates in 200 μL serum-free RPMI medium in the upper well. Complete medium was added to the lower well. After 24 h of incubation, cells that migrated through the membrane to the lower well were all counted by light microscopy.

### Immunofluorescence

Cells cultured on cover glass slides (Menzel-Gläser; Braunschweig, Germany) were fixed and permeabilized with Cytofix-Cytoperm solution (BD Biosciences, Le Pont de Claix, France) for 10 min at room temperature. Cells were washed with 2% glycine solution, then blocked with blocking buffer containing 2% FBS and 0.5% saponin in PBS for 30 min at 37 °C. Cells were incubated with the primary antibodies, then with Alexa Fluor-conjugated secondary antibodies or phalloidin for 1 h at room temperature each. DAPI was added for 2–3 min at room temperature. The slides were mounted with Mowiol solution (Southern Biotech, Birmingham, AL). Immunofluorescence images were acquired with inverted epifluorescence or confocal microscopes and analyzed with IMSTAR or LEICA softwares, respectively.

### Enzyme-linked immunosorbent assay (ELISA)

Soluble E-cadherin was detected in conditioned media from shRNA lentiviral transduced cells with sandwich ELISA (Human E-cadherin EIA Kit, Takara Bio, Osaka, Japan), according to the manufacturer’s specifications. Briefly, samples were added, in triplicates, to the wells and then incubated in the dark for 2 h at 37 °C. The wells were washed 3 times with TBS containing 0.1% Tween-20 and 5 mM CaCl_2_. The antibody conjugated to peroxidase and specific to human E-cadherin was added for 1 h at 37 °C, the wells were washed 4 times and then the substrate solution (TMBZ) was added for 15 min at room temperature before the reaction was stopped with 1 N sulfuric acid. The plate was read at 450 nm.

## Results

### Comparative analysis of E- and N-cadherin expression and oncogenic properties between primary melanoma cells and their respective metastatic cells

To study the possibility to revert the E- to N-cadherin switch and the tumor phenotype in metastatic melanoma, the appropriate cellular model (i.e. aggressive metastasis-derived melanoma cells that do not express E-cadherin but strongly express N-cadherin) was first selected. Therefore, the expression of these cadherins and the oncogenic properties (i.e. anchorage-dependent and -independent growth and migration) were analyzed in two couples of primary and corresponding lymph-node metastasis melanoma cells. I5 (primary tumor) and M2 (lymph-node metastasis) cell lines were derived from the same patient. T1 (primary tumor) and G1 (lymph-node metastasis) cell lines were derived from another patient.

**Fig. 1:**
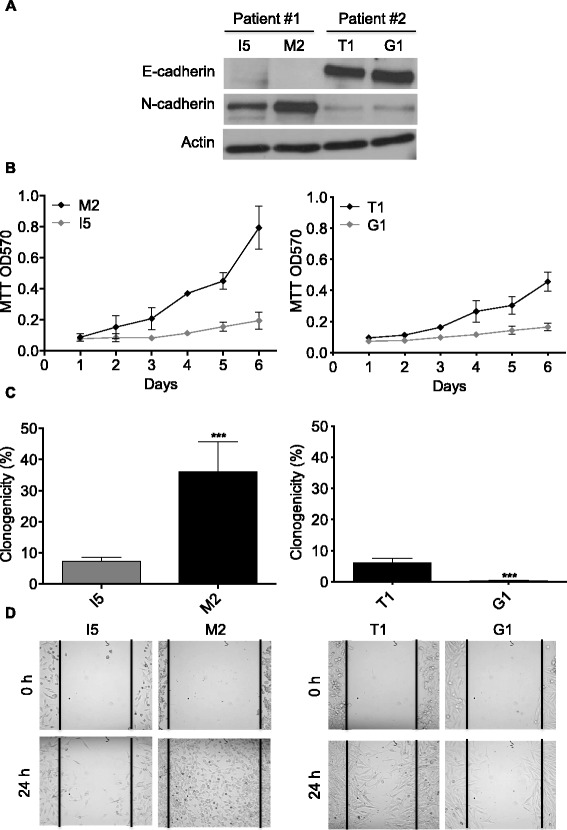
Comparative analysis of E- and N-cadherin expression and oncogenic properties between primary tumor and respective metastatic melanoma cell lines **a**. Protein extracts from the primary tumor (I5 and T1) and respective lymph-node metastatic (M2 and G1) melanoma cell lines were analyzed by western blot using anti-E-cadherin, anti-N-cadherin and anti-actin antibodies. The results presented are those of typical experiments. **b**. The proliferation of I5, M2, T1 and G1 cells was analyzed over six days by MTT assay. Results presented are the means ± SD for three independent experiments. **c**. I5, M2, T1 and G1 cells were cultured in methylcellulose semi-solid medium for three weeks. Then, the colonies formed were counted. The results are presented as the percentage of seeded cells that form colonies (clonogenicity) and are the means ± SD for three independent experiments. ***, *p* < 0.005 versus respective primary tumor cells. **d**. Representative images of cell migration evaluated by wound healing assay. Images of wound repair were taken at 0 and 24 h after wound

The expression of E- and N-cadherins in I5, M2, T1 and G1 melanoma cell lines was analyzed by western blot (Fig. 1a). High expression levels of E-cadherin and very low expression levels of N-cadherin were found in T1 and G1 cells. On the other hand, both I5 and M2 cells were found to express N-cadherin but not E-cadherin. Specifically, the metastatic M2 cells showed a significant 5-fold increase in N-cadherin expression level in comparison to their corresponding primary tumor cells (I5). These results suggest that the I5 and M2 melanoma cells arise from melanocyte(s) that underwent the cadherin switch.

The proliferation (Fig. 1b), anchorage-independent growth (Fig. 1c) and migration (Fig. 1d) of the melanoma cell lines were also analyzed. Except for the I5 cell line, these mesenchymal features strongly and significantly correlate with high N-cadherin and low E-cadherin expression in our set of melanoma models (Additional file 1). Furthermore, significantly higher proliferation, anchorage-independent growth (anoikis resistance) and migration rates were found in M2 cells that don’t express E-cadherin but express the highest level of N-cadherin compared to I5, T1 and G1 cells. Thus, the M2 cell line is the most aggressive among this set of melanoma models.

Taken together, these results suggest that the highly aggressive E-cadherin-negative/N-cadherin-positive metastatic M2 cell line is an appropriate cellular model to study the possibility to induce the reversion of the cadherin switch and tumor phenotype in aggressive metastatic melanoma.

### Effect of Gö6976 and Gö6983 on E- and N-cadherin expression in M2 metastatic melanoma cells

**Fig. 2:**
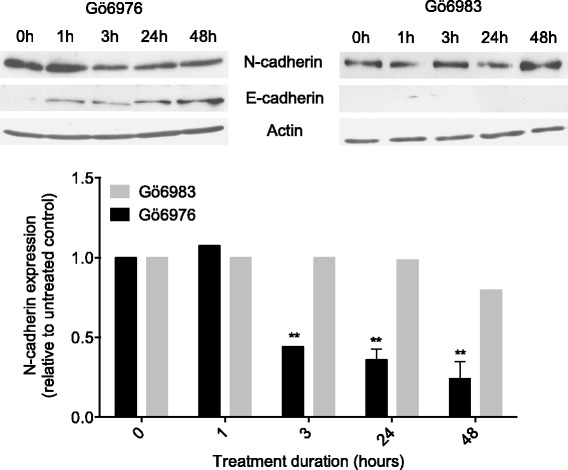
Gö6976 but not Gö6983 inhibits N-cadherin expression and induces E-cadherin expression in M2 metastatic melanoma cells. M2 metastatic melanoma cells were seeded in 6-well plates at the density of 25,000 cells per well. After three to five days of culture, cells were treated with 1 μM Gö6976 or 1 μM Gö6983 for different periods of time (0, 1, 3, 24 or 48 h). Cells were then lysed and proteins analyzed by western blot using anti-E-cadherin, anti-N-cadherin or anti-actin antibodies. The autoradiograms presented are those of a typical experiment. The histogram represents quantitative analysis of N-cadherin expression normalized to untreated control cells (0 h). The results presented are the means ± SD for three independent experiments. **, *p* < 0.01 versus untreated cells

To determine whether PKC inhibitors may affect the expression of E- and/or N-cadherin in metastatic melanoma cells, M2 cells were incubated with 1 μM Gö6976 or 1 μM Gö6983 for different periods of time (0, 1, 3, 24 and 48 h), then analyzed by western blot (Fig. 2). Compared to untreated control cells (0 h), Gö6976-treated cells exhibited an induced expression of E-cadherin and a significantly decreased (by approximately 50%) expression of N-cadherin as soon as 1 h and 3 h of treatment, respectively. This Gö6976-induced N- to E-cadherin switch was maintained for at least 48 h. However, treatment of the M2 cells with Gö6983 affected neither N-cadherin nor E-cadherin expression. These results demonstrate that Gö6976 but not Gö6983 induces E-cadherin and reduces N-cadherin expression in M2 metastatic melanoma cells.

### Effect of Gö6976 and Gö6983 on the morphology and intercellular interactions of M2 metastatic melanoma cells

**Fig. 3:**
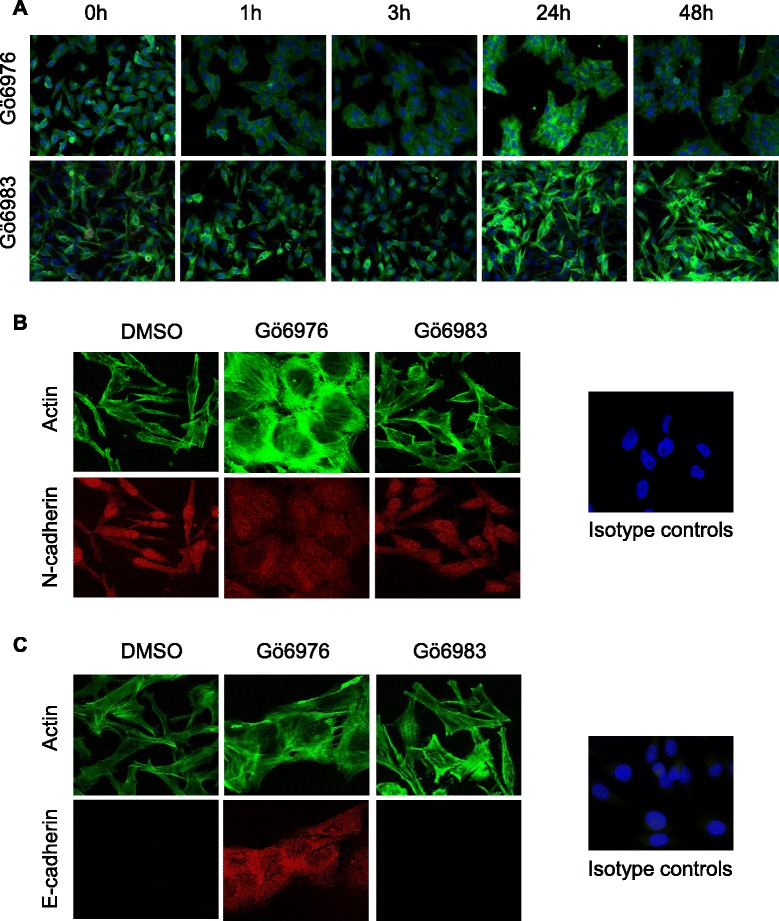
Gö6976 but not Gö6983 induces rapid cell shape modification and E-cadherin-associated cell-cell interactions in M2 mesenchymal-like metastatic melanoma cells. M2 metastatic melanoma cells were seeded in 6-well plates at the density of 12,500 cells per well. After three to five days of culture, cells were treated with 1 μM Gö6976, 1 μM Gö6983 or vehicle (DMSO) for different periods of time (0, 1, 3, 24 or 48 h) (**a**) or for 24 h only (**b** and **c**). Cells were then fixed, permeabilized and stained for actin (green) alone (**a**), co-stained for actin and N-cadherin (*red*) (**b**) or co-stained for actin and E-cadherin (*red*) (**c**). Isotype-matched control antibodies were used. Cell nuclei were stained with DAPI (*blue*). Cells were analyzed under epifluorescence (**a**) or confocal (**b** and **c**) microscope. Images are representative fields from three or more independent experiments

The cadherin switch is a major event of the EMT during tumor progression that entails profound morphological changes to a cell and affects intercellular interactions [16]. Therefore, the effect of the PKC inhibitors (Gö6976 and Gö6983) on the M2 cell morphology and intercellular interactions was analyzed by actin labeling and fluorescent microscopy (Fig. 3a). Untreated cells (0 h) showed a typical elongated mesenchymal-like shape and formed very few cell-cell contacts. Treatment with Gö6976 induced a rapid cell shape change from elongated to cuboidal accompanied by a strong increase in cell-cell interactions (Fig. 3a). These changes were observed as early as 1 h after treatment with Gö6976 and were maintained for at least 48 h. In contrast, Gö6983-treated cells remained elongated and isolated (Fig. 3a). Thus, Gö6976 but not Gö6983 induces a rapid cell shape modification from an elongated mesenchymal-like structure into a “cuboidal” epithelial-like shape and a strong increase in intercellular interactions in M2 metastatic melanoma cells.

### Cellular localization of the Gö6976-induced E-cadherin molecules

Since Gö6976 was found to regulate the expression of the cell adhesion molecules N- and E-cadherins as well as the intercellular interactions of M2 cells (Figs. 2 and 3a), the subcellular localization of these cadherins was next analyzed. Therefore, M2 cells were treated with Gö6976 or Gö6983 for 24 h before actin and N-cadherin (Fig. 3b) or E-cadherin (Fig. 3c) fluorescent co-staining and confocal microscopy analysis. Whatever the condition, N-cadherin staining was mostly localized in the cytoplasm (Fig. 3b). On the other hand, E-cadherin staining was observed only in Gö6976-treated, but not in untreated or Gö6983-treated cells and was strongly increased at the cell-cell contacts (Fig. 3c). Thus, Gö6976-incited cell clustering is associated with induced E-cadherin expression and localization at the intercellular interactions.

### Effect of Gö6976 and Gö6983 on the subcellular localization of β-catenin

**Fig. 4:**
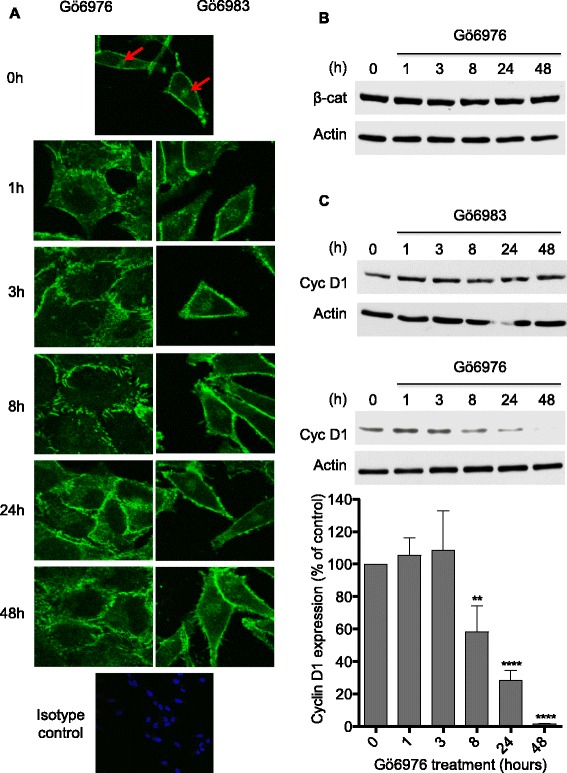
Gö6976 but not Gö6983 induces rapid β-catenin translocation outside of the nucleus and reduces cyclin D1 expression in M2 metastatic melanoma cells. M2 metastatic melanoma cells were seeded in 6-well plates at the density of 12,500 cells per well. After three to five days of culture, cells were treated with 1 μM Gö6976 or 1 μM Gö6983 for different periods of time (0, 1, 3, 8, 24 or 48 h). **a**. After treatment, cells were fixed, permeabilized, stained for β-catenin (*green*) and analyzed under a confocal microscope. Isotype-matched control antibody and DAPI (*blue*) were used. Images are representative fields from three or more independent experiments. **b**. After treatment cells were lysed and proteins analyzed by western blot using anti-β-catenin or anti-actin antibodies. The autoradiograms presented are those of a typical experiment. **c**. After treatment cells were lysed and proteins analyzed by western blot using anti-cyclin D1 or anti-actin antibodies. The autoradiograms presented are those of a typical experiment. The histogram represent quantitative analysis of cyclin D1 expression following Gö6976 treatment normalized to untreated control cells (0 h). The results presented are the means ± SD for three independent experiments. **, *p* < 0.01; ****, *p* < 0.0001 versus untreated cells

The stability of E-cadherin-mediated intercellular interactions requires the accumulation of β-catenin at the plasma membrane at these cell-cell contact sites [31]. Loss of E-cadherin expression during EMT and tumor progression is accompanied by β-catenin translocation to the nucleus where it acts as oncogenic transcription factor [32, 33]. Since Gö6976-treatment induces the expression of E-cadherin at the cellular junctions in the E-cadherin-negative metastatic melanoma cells (M2 cells), the status of β-catenin in these cells and the effect of the PKC inhibitors (Gö6976 and Gö6983) on its subcellular localization and expression were determined (Fig. 4a and b). In untreated M2 mesenchymal-like melanoma cells (0 h), β-catenin was accumulated at high levels in the nuclei of the cells in addition to its location at the plasma membrane (Fig. 4a). When the cells were treated with Gö6976, β-catenin staining disappeared from the nuclei as soon as 1 h of treatment and was observed at the plasma membrane (Fig. 4a, left column). This Gö6976-induced relocation of β-catenin from the nucleus to the plasma membrane was stable and maintained for at least 48 h of treatment (Fig. 4a, left column). Noteworthy, Gö6976 did not affect β-catenin expression (Fig. 4b). On the other hand, when the cells were treated with Gö6983, β-catenin was still located at high levels in the nuclei of the cells whatever the duration of treatment (1–48 h) (Fig. 4a, right column). In conclusion, Gö6976, but not Gö6983, induces the translocation of β-catenin from the nucleus to the plasma membrane.

### Effect of Gö6976 on cyclin D1 expression

To determine whether the Gö6976-induced relocation of β-catenin from the nucleus to the plasma membrane was associated with a decrease in its oncogenic function, the effect of Gö6976 treatment on the expression of a major β-catenin target, i.e. the oncoprotein cyclin D1, was analyzed (Fig. 4c). M2 cells were incubated with Gö6976 or Gö6983 for different periods of time then lysed and cyclin D1 expression analyzed by western blot using a specific antibody. As shown in Fig. 4a, cyclin D1 was strongly expressed in untreated M2 cells (0 h). Gö6976 treatment markedly inhibited cyclin D1 expression levels over time; by approximately 41% and 93% after 8 and 48 h of treatment, respectively. Thus, Gö6976 treatment inhibits cyclin D1 expression in M2 melanoma cells. In contrast to Gö6976, Gö6983 didn’t have any significant effect on cyclin D1 expression. These data further confirm that the reduction in cyclin D1 by Gö6976 may reflect changes in cadherin expression patterns.

### Effect of Gö6976 and Gö6983 on the metastatic and migration potential of M2 melanoma cells

**Fig. 5:**
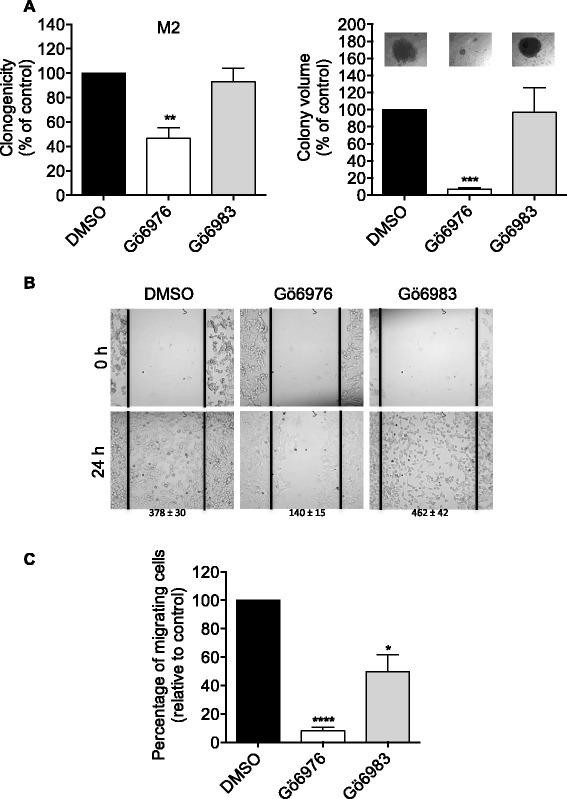
Gö6976 but not Gö6983 strongly inhibits anchorage-independent growth and migration of M2 metastatic melanoma cells. **a**. M2 metastatic melanoma cells were seeded in methylcellulose (400 cells per plate) containing 1 μM Gö6976 or 1 μM Gö6983. After three weeks of culture, the colonies were counted, photographed and their volume determined. The quantifications presented are the means ± SD for three independent experiments. **, *p* < 0.01; ***, *p* < 0.001 versus control DMSO treated cells. **b**. Scratch wounds were created by scraping confluent monolayers of M2 cells, pre-treated one day before with 1 μM Gö6976, 1 μM Gö6983 or DMSO. After 24 h, cell migration from the wound edges into the wounded area was evaluated. Cells were photographed at 0 and 24 h after wound. The photographs presented are those of a representative experiment. Values presented under each photograph represent the mean ± SD of numbers of cells that migrated into the wound for three independent experiments. **c**. Cells were incubated in RPMI medium containing 1 μM Gö6976, 1 μM Gö6983 or DMSO in the upper well of a transwell chamber while in the lower well RPMI medium supplemented with 10% FBS was added. After 24 h, cells that invaded to the lower well were counted. The results presented are the means ± SD for three independent experiments. *, *p* < 0.05; ****, *p* < 0.0001 versus control DMSO treated cells

Loss of E-cadherin expression, nuclear localization of β-catenin and cyclin D1 expression are characteristics that have been shown to correlate with transformation, increased oncogenic activity and progression of most cancers, including melanoma [9, 34]. To test whether the Gö6976-induced reversion of these characteristics (Figs. 1, 2, 3 and 4) correlates with a reduced aggressivity of the metastatic melanoma cells, the effect of this inhibitor on the anchorage-independent growth (reflecting cell metastatic potential) and migration capacity of the M2 cells was determined (Fig. 5).

Anchorage-independent growth was analyzed by studying the ability of M2 cells treated or not with Gö6976 or Gö6983 to survive, grow and form colonies in the methylcellulose semi-solid medium. As shown in Fig. 5a, Gö6976 strongly reduced the number and volume of the colonies formed in methylcellulose (by 54% and 93.2% respectively), whereas Gö6983 had no significant effect. Gö6976 also strongly inhibited by 62.9% (Fig. 5b) and 91.9% (Fig. 5c) the horizontal (wound healing assay) and chemotactic (transwell assay) migrations, respectively.

Noteworthy, although the horizontal migration was not affected by Gö6983 (Fig. 5b), this inhibitor significantly reduced, by 50%, the chemotactic migration of M2 cells but to a lesser extent than Gö6976 (percentage of inhibition = 93.2%) (Fig. 5c).

Together, these results indicate that the Gö6976-induced E-cadherin expression, membrane translocation of β-catenin and loss of cyclin D1 expression correlate with a decreased anchorage-independent growth and migration capacity of the M2 metastatic melanoma cells.

### Effect of Gö6976 and Gö6983 on the metastatic and migration potential of M4T2 melanoma cells

To further confirm the ability of Gö6976 to revert the E- to N-cadherin switch and pro-metastatic phenotype of aggressive melanoma cells, the effect of this inhibitor was also tested on M4T2 cells that are derived from the cutaneous metastasis of a different melanoma patient and express high levels of N-cadherin but low levels of E-cadherin.

**Fig. 6:**
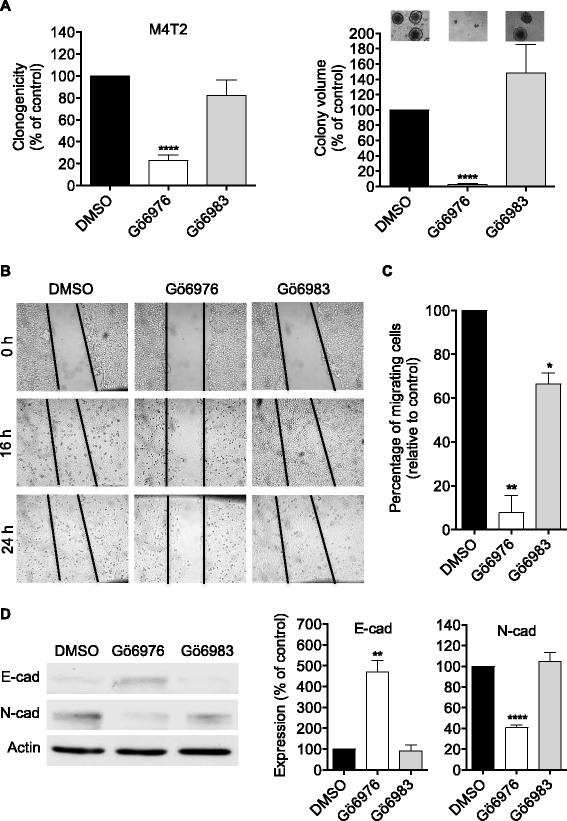
Gö6976 but not Gö6983 strongly inhibits anchorage-independent growth and migration and reverses the E to N-cadherin switch in M4T2 metastatic melanoma cells. **a**. M4T2 metastatic melanoma cells were seeded in methylcellulose (1000 cells per plate) containing 1 μM Gö6976 or 1 μM Gö6983. After three weeks of culture, the colonies were counted, photographed and their volume determined. The quantifications presented are the means ± SD for three independent experiments. ****, *p* < 0.0001 versus control DMSO treated cells. **b**. Scratch wounds were created by scraping confluent monolayers of M4T2 cells, pre-treated one day before with 1 μM Gö6976, 1 μM Gö6983 or DMSO. After 16 and 24 h, cell migration from the wound edges into the wounded area was evaluated. Cells were photographed at 0, 16 and 24 h after wound. The photographs presented are those of a representative experiment. **c**. Cells were incubated in RPMI medium containing 1 μM Gö6976, 1 μM Gö6983 or DMSO in the upper well of a transwell chamber while in the lower well RPMI medium supplemented with 10% FBS was added. After 24 h, cells that invaded to the lower well were counted. The results presented are the means ± SD for three independent experiments. *, *p* < 0.05; **, *p* < 0.01 versus control DMSO treated cells. **d**. Cells were seeded in 6-well plates at the density of 25,000 cells per well. After three to five days of culture, cells were treated with 1 μM Gö6976, 1 μM Gö6983 or DMSO for 24 h). Cells were then lysed and proteins analyzed by western blot using anti-E-cadherin, anti-N-cadherin or anti-actin antibodies. The autoradiograms presented are those of a typical experiment. The histograms represent quantitative analysis of E- or N-cadherin expression normalized to control DMSO treated cells. The results presented are the means ± SD for two independent experiments. **, *p* < 0.01; ****, *p* < 0.0001 versus DMSO treated cells

As shown in Fig. 6, treatment of M4T2 melanoma cells with Gö6976 strongly inhibits anchorage-independent growth (approximately 76.9% and 97.1% reduction in the number and volume of colonies, respectively; Fig. 6a), horizontal migration (very faint wound closure 24 h after scratch while full closure observed after at least 16 h for control DMSO-treated cells; Fig. 6b) and chemotactic migration (by approximately 92%; Fig. 6c).

Furthermore, treatment of M4T2 cells with Gö6976 also induced a 4.7-fold increase in E-cadherin expression and a decrease in N-cadherin expression by 40.8% (Fig. 6d) compared to control (DMSO-treated) cells.

On the other hand, treatment of M4T2 cells with Gö6983 reduced their chemotactic migration by only 33.6% (vs. 92% when treated with Gö6976) (Fig. 6c), and did not affect horizontal migration (Fig. 6b), anchorage-independent growth (Fig. 6a) or cadherin switch (Fig. 6d).

Together, these results show that in the same way as in M2 cells, treatment with Gö6976, but not with Gö6983, induces N- to E-cadherin switch and reversion of the pro-metastatic phenotype in the M4T2 metastatic melanoma cells.

In contrast to the metastatic melanoma cells (M2 and M4T2 cell lines) (Figs. 2, 5a and 6a, d), Gö6976 inhibitor did not significantly affect the expression of E- or N-cadherins or the anchorage-independent growth in the primary melanoma cell line (I5) (Additional file 2). Thus, the effects of this inhibitor seem to be specific to advanced metastatic melanoma.

Taken all together, our results have shown that the PKC inhibitors; i.e. Gö6976 and Gö6983, differentially affect the reversion of E- to N-cadherin switch and metastatic phenotype in aggressive melanoma cells (Figs. 1, 2, 3, 4, 5 and 6). In fact, whereas Gö6976 induces E-cadherin expression, β-catenin translocation from the nucleus to the plasma membrane and intercellular interactions, and inhibits N-cadherin and cyclin D1 expression, anchorage-independent growth and horizontal and chemotactic migration of metastatic mesenchymal-like melanoma cells, Gö6983 could only reduce the chemotactic migration but to a much lesser extent than Gö6976. These results suggest that a specific PKC isoform targeted by Gö6976 but not by Gö6983 would be involved in the reversion of the cadherin switch and metastatic phenotype and; therefore, would become a pharmaceutical target for the treatment of melanoma. Next, we searched to determine this target.

### Analysis of PKD1 expression in different melanoma cell lines and correlation with E-/N-cadherin expression

Since Gö6976 is a selective inhibitor of PKCα, PKCβ and PKCμ and Gö6983 a selective inhibitor of PKCα and PKCβ but not PKCμ [26, 27], we hypothesized that the specific inhibition of PKCμ, which is also known as PKD1 (protein kinase D1), would be responsible for the Gö6976-induced N- to E-cadherin switch and tumor reversion in metastatic melanoma cells.

**Fig. 7:**
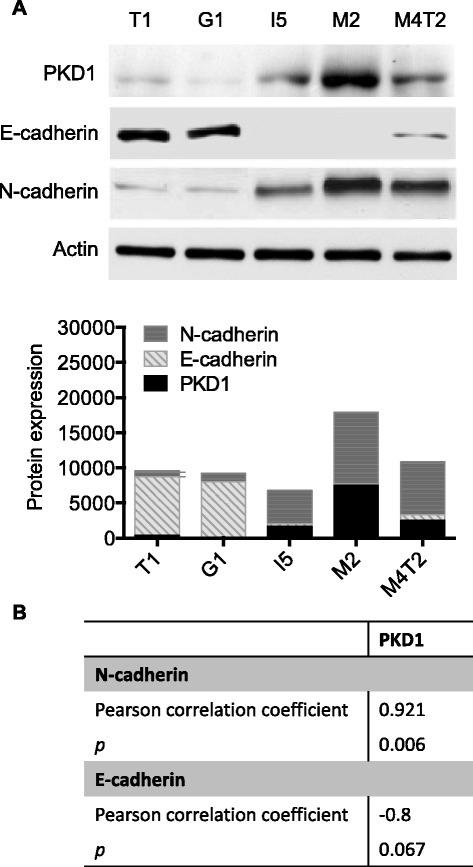
Protein kinase D1 expression positively correlates with N-cadherin and negatively correlates with E-cadherin in primary and metastatic melanoma cells. Protein extracts from primary (T1 and I5) and metastatic (G1, M2 and M4T2) melanoma cells were analyzed by western blot using anti-PKD1, anti-E-cadherin, anti-N-cadherin and anti-actin antibodies. **a**. The results presented are those of typical experiments. The histograms represent quantitative analysis of PKD1, N-cadherin and E-cadherin expression from two independent experiments. **b**. The table represents the relationship between PKD1 protein levels and E-cadherin and N-cadherin levels in the melanoma cell lines

To test this hypothesis, first, the expression of PKD1 and its relationship with E- and N-cadherin statuses were analyzed by western blot in different melanoma cell lines (i.e. T1, G1, I5, M2 and M4T2) (Fig. 7). In T1 and G1 melanoma cells that express high levels of E-cadherin but very low levels of N-cadherin, PKD1 expression was very faint (Fig. 7a). In I5, M2 and M4T2 melanoma cells, that express null or very low levels of E-cadherin but high levels of N-cadherin, PKD1 expression was strong with maximal expression in the most aggressive cell line (i.e. M2 cells) (Fig. 7a). In addition to this strong qualitative correlation between PKD1 expression and E-cadherin negative/N-cadherin positive phenotype, quantitative correlation was also confirmed by Spearman correlation coefficient analysis (*r* = 0.921; *p* = 0.006 and *r* = −0.8; 0.067 for correlation with N-cadherin and E-cadherin, respectively) (Fig. 7b). Furthermore, PKD1 expression significantly correlated with the mesenchymal features of the melanoma cell lines used in this study and was associated with a high metastatic potential (anchorage-independent growth and migration) (Additional file 3).

These data support the hypothesis that the effect of Gö6976 on N- to E-cadherin switch and tumor reversion in melanoma cells would be due to the inhibition of PKD1.

### PKD1 silencing by lentiviral vector based shRNA transduction in the M2 metastatic melanoma cells

**Fig. 8:**
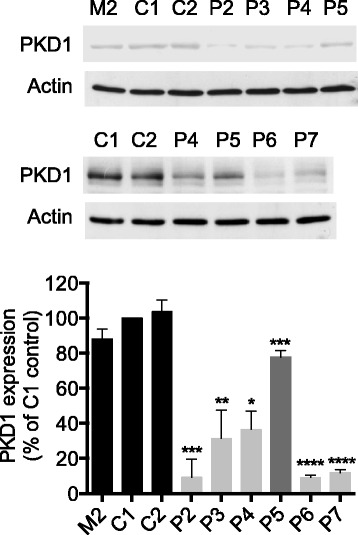
PKD1 silencing by lentiviral vector based shRNA transduction in the M2 metastatic melanoma cells. Protein extracts from parental (M2), lentiviral PKD1-specific (clones P2, P3, P4, P5, P6 and P7) and control (clones C1 and C2) shRNA-transduced M2 cells were analyzed by western blot using anti-PKD1 and anti-actin antibodies. The results presented are those of typical experiments. The histograms represent quantitative analysis of PKD1 expression normalized to C1 control clone. The results presented are the means ± SD for two independent experiments. *, *p* < 0.05; **, *p* < 0.01; ***, *p* < 0.001; ****, *p* < 0.0001 versus C1 and C2 control clones

Next, to allow direct analysis of the role of PKD1 inhibition in human metastatic melanoma, M2 cell clones stably expressing shRNAs directed against PKD1 (named P2, P3, P4, P5, P6 and P7) or scrambled shRNA control (named C1 and C2) were generated by lentiviral particles transduction. The efficiency of PKD1 knockdown was determined by western blot (Fig. 8). The results show that whereas the clones transduced with lentiviral control shRNA express similar levels of PKD1 protein as parental M2 cells, clones transduced with lentiviral PKD1-specific shRNAs show varying degrees of PKD1 knockdown efficiency. PKD1 expression was strongly inhibited in P6, P2, P7, P3 and P4 clones by approximately 90.9, 90.78, 88.1, 68.7 and 63.5%, respectively, but weakly inhibited in P5 clone by approximately 20%.

### Effect of PKD1 depletion on E-cadherin expression in M2 mesenchymal-like melanoma cells

**Fig. 9:**
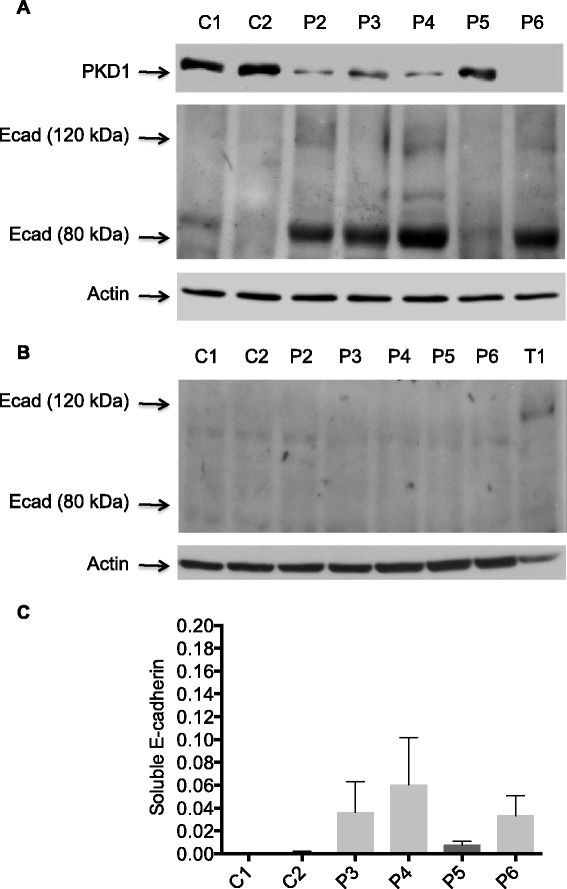
The E-cadherin induced by PKD1 depletion is cleaved in M2 metastatic melanoma cells. **a**. Protein extracts from control (C1 and C2 clones) and PKD1 depleted (P2, P3, P4, P5, P6 and P7 clones) M2 cells cultured in methylcellulose semi-solid medium for three weeks were analyzed by western blot using anti-PKD1, anti-E-cadherin and anti-actin antibodies. The results presented are those of typical experiments. **b**. Protein extracts from control (C1 and C2 clones) and PKD1 depleted (P2, P3, P4, P5, P6 and P7 clones) M2 cells cultured in adherent conditions were analyzed by western blot using anti-E-cadherin and anti-actin antibodies. T1 melanoma cells were used as positive control for E-cadherin expression. The results presented are those of typical experiments. **c**. Conditioned media from control (C1 and C2) and PKD1 depleted (P3, P4, P5 and P6) M2 cells were analyzed for E-cadherin by ELISA

Since Gö6976 but not Gö6983 induces E-cadherin expression in the E-cadherin-negative metastatic melanoma cells (Fig. 2), we tested whether PKD1 depletion would have the same effect. Therefore, total proteins from lentiviral control (C1 and C2) and PKD1-specific (P2, P3, P4, P5 and P6) shRNA-transduced M2 clones cultured in semi-solid methylcellulose medium (Fig. 9a) or in adherent conditions (Fig. 9b) were extracted and analyzed for PKD1 and/or E-cadherin expression by western blot. A very faint protein band at 120 kDa and a very thick band at 80 kDa representing full-length and cleaved E-cadherin molecules, respectively, were observed in the protein extracts from PKD1-depleted (P2, P3, P4 and P6) M2 clones cultured in methylcellulose but not from control (C1 and C2) or PKD1-faintly inhibited (P5) M2 clones (Fig. 9a). Nonetheless, when these cells were cultured under adherent conditions (Fig. 9b), neither full-length nor cleaved E-cadherin corresponding bands could be observed in the protein extracts from any of the M2 clones (Fig. 9b).

Since a strong expression of cleaved E-cadherin was observed in PKD1-depleted M2 clones cultured in methylcellulose, we asked whether induced E-cadherin would be cleaved and secreted in the medium of the adherent cells while sequestered in the spheres of colonies formed in methylcellulose. To test this hypothesis, conditioned media from control (C1 and C2) and PKD1-specific (P3, P4, P5 and P6) shRNA-transduced M2 clones were analyzed for the presence of soluble E-cadherin by ELISA. As indicated in Fig. 9c, significant amounts of soluble E-cadherin were detected in the media of PKD1-specific (P3, P4, P5 and P6) but not control (C1 and C2) shRNA-transduced M2 clones. Taken together, these results indicate that PKD1 depletion induces the expression of E-cadherin that is cleaved and secreted in M2 cells.

Interestingly, PKD1 knockdown also induced the expression of E-cadherin in M4T2 metastatic melanoma cells. However, in contrast to the M2 cells, full-length E-cadherin was detected in M4T2 cells (Additional file 4). This result further confirms the role of PKD1 in the regulation of E-cadherin expression and also suggests that the mechanism leading to the cleavage of the newly formed E-cadherin molecules in M2 cells is cell-context specific.

### Effect of PKD1 depletion on the cell shape and cell-cell interactions in M2 melanoma cells

**Fig. 10:**
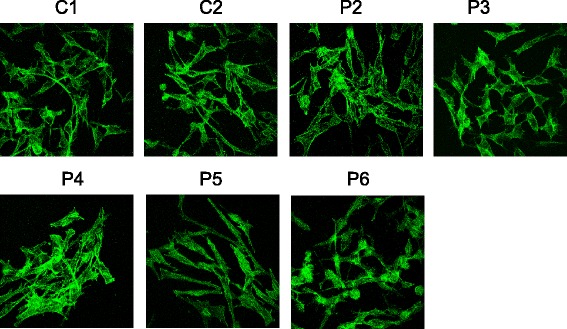
PKD1 depletion does not affect the cell shape or cell-cell interactions in M2 mesenchymal-like metastatic melanoma cells. M2 melanoma cells were seeded in 6-well plates at the density of 12,500 (control C1, C2 and P5 clones) or 25,000 (PKD1-depleted P2, P3, P4 and P6 clones) cells per well. After three to five days of culture, cells were fixed, permeabilized and stained for actin (*green*). Cells were then analyzed under a confocal microscope. Images are representative fields from three independent experiments

Since Gö6976 but not Gö6983 induces a rapid cell shape modification (from elongated to cuboidal) and cell-cell interactions in M2 cells (Fig. 3a), we tested whether PKD1 depletion may exert the same effect. Therefore, the cell morphology and intercellular interactions of control (C1 and C2) and PKD1-specific (P2, P3, P4, P5, P6 and P7) shRNA-transduced M2 clones were analyzed by actin-labeling and confocal microscopy (Fig. 10). Results showed that all shRNA-transduced M2 clones (control and PKD1-depleted) present typical elongated mesenchymal-like shape and form very few cell-cell contacts, suggesting that PKD1 depletion could induce neither reversion of mesenchymal-like morphology nor intercellular interactions in M2 cells.

### Effect of PKD1 silencing on the metastatic potential of M2 melanoma cells

**Fig. 11:**
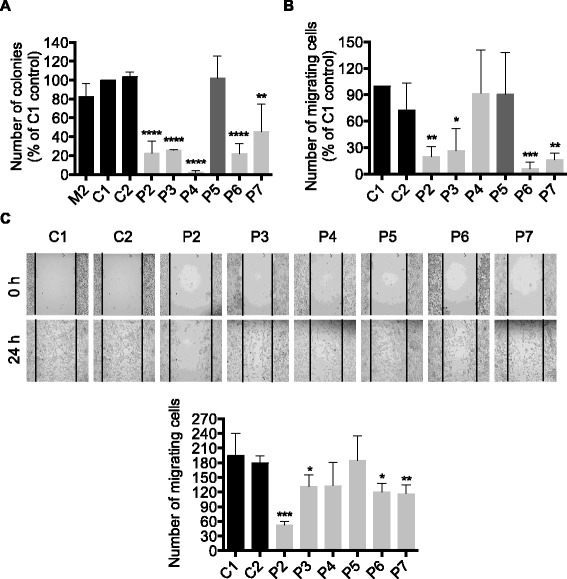
PKD1 depletion inhibits anchorage-independent growth and migration of M2 metastatic melanoma cells. **a**. PKD1 depleted (P2, P3, P4, P5, P6 and P7 clones) and control (C1 and C2 clones) M2 cells were cultured in methylcellulose semi-solid medium for three weeks. Then, the colonies formed were counted. The quantifications presented are the means ± SD for three independent experiments. **, *p* < 0.01; ****, *p* < 0.0001 versus C1 and C2 control cells. **b**. Cells were incubated in RPMI medium in the upper well of a transwell chamber while in the lower well RPMI medium supplemented with 10% FBS was added. After 24 h, cells that invaded to the lower well were counted. The results presented are the means ± SD for three independent experiments. *, *p* < 0.05; **, *p* < 0.01; ***, *p* < 0.001 versus C1 and C2 control cells. **c**. Representative images of cell migration evaluated by wound healing assay. Images of wound repair were taken at 0 and 24 h after wound and the cells that migrated to the wounded area were counted. The results presented are the means ± SD for three independent experiments. *, *p* < 0.05; **, *p* < 0.01 versus C1 and C2 control cells

To determine whether PKD1 inhibition may affect the metastatic potential of M2 melanoma cells, control (C1 and C2) and PKD1-specific (P2, P3, P4, P5, P6 and P7) shRNA-transduced M2 clones were assayed for anchorage-independent growth and migration. Results show that anchorage-independent growth of PKD1-depleted (i.e. P2, P3, P4, P6 and P7) M2 clones was strongly inhibited by 77.5, 74.1, 97.8, 78 and 54.5%, respectively, in comparison to control shRNA-transduced (C1 and C2) and parental (M2) cells (Fig. 11a). Furthermore, except for P4 clone, chemotactic migration of all other PKD1-depleted (P2, P3, P6, P7) M2 clones was strongly inhibited by 80.4, 73.6, 93.7 and 84%, respectively (Fig. 11b). However, horizontal (wound healing) migration was not significantly (P4) or weakly inhibited by 27% (P3), 33% (P6) and 35% (P7) compared to control shRNA-transduced (C1 and C2) cells, in all PKD1-depleted clones, except for P2 clone (strong inhibition by 70.5%) (Fig. 11c).

Anchorage-independent growth and migration rates of P5 clone, in which PKD1 expression was only slightly inhibited, were similar to control cells (Fig. 11) suggesting that a specific and strong PKD1 inhibition is required to revert the metastatic phenotype in melanoma cells.

## Discussion

In this study, we tested whether PKC inhibitors (such as Gö6976 and Gö6983) would revert the E- to N-cadherin switch, a major hallmark of the EMT process, required for melanoma progression and metastasis [9, 15, 35].

First, we analyzed the cadherin switch in two couples of patient-derived primary (I5, T1) and their respective lymph-node metastasis (M2, G1) melanoma cells. Different profiles of E- and N-cadherin expression were observed. When comparing T1 (primary tumor) and G1 (corresponding lymph node metastasis) melanoma cells, both cell lines expressed high levels of E-cadherin and very low levels of N-cadherin. Although these findings are inconsistent with the theory of E- to N-cadherin switch during melanoma progression (i.e. E-cadherin expression in primary tumor and N-cadherin expression in metastasis), the expression of E-cadherin by the metastasis-derived cells (G1) might be the consequence of a MET (mesenchymal-epithelial transition) that would have occurred at the metastatic site. MET was postulated to be part of the process of metastatic tumor formation [36, 37]. In fact, progression of solid tumors involves spatial and temporal occurrences of EMT, whereby tumor cells acquire a more invasive and metastatic phenotype [38]. Subsequently, the disseminated mesenchymal tumor cells undergo the reverse transition, MET, at the site of metastases, as metastases recapitulate the pathology of their corresponding primary tumors [39]. Just as a critical EMT event is the downregulation or silencing of E-cadherin and upregulation of N-cadherin, the re-expression of E-cadherin and loss of N-cadherin is proposed to be the important hallmark of MET [39]. Presently, it is unclear how MET facilitates the formation of metastases. Some findings suggest a possible mechanism whereby MET helps the tumor cells to construct connections with the resident non-neoplastic epithelial cells and may enable dormancy and survival of tumor cells at a lower metabolic load at target organ [39]. Consistently, G1 cells, that may be derived from metastatic cells that have underwent MET, grow very slowly, are non-tumorigenic and do not migrate. On the other hand, E-cadherin expression was absent in both I5 (primary tumor) and M2 (corresponding lymph node metastasis) melanoma cells while N-cadherin expression was significantly higher in the metastasis cells, suggesting that I5 primary tumor cells may have already underwent EMT. However, these cells did not show higher proliferation and migration potentials than their counterparts T1 cells but progressed to highly aggressive metastatic cells (i.e. M2 cells). This observation suggests that depending on the cellular context, the pro-metastatic effect of cadherin switch might require additional events that could occur later in tumorigenesis [16]. In agreement with this hypothesis, a study by Chen et al. 2014 shows that E-cadherin loss in non-malignant breast cells is insufficient to induce EMT or to enhance their transforming potential [40]. Furthermore, other studies by Knudsen et al. 2005 show that N-cadherin expression in the mammary gland of transgenic mice (mice expressing N-cadherin under the control of mouse mammary tumor virus promoter) does not induce tumors. Interestingly, even crossing these mice with mice expressing the Neu oncogene in the mammary gland does not produce tumors more aggressive than those in mice expressing Neu alone [41]. However, crossing them with mice expressing polyoma virus middle T antigen in the mammary epithelium leads to increased metastasis [42]. These studies suggest that the effects of cadherin switching are late events in tumorigenesis and demonstrate that the influence of an inappropriate cadherin on the phenotype of the cell is context dependent [16]. This could be the same in melanoma cells. Thus, despite the E- to N-cadherin switch in I5 primary melanoma cells, these cells require additional mutations or events to acquire enhanced mesenchymal features allowing their invasion and metastasis formation. The primary (I5) and corresponding metastasis (M2) melanoma cells could be a good model to study the events that are required in addition to the E- to N-cadherin switch to allow melanoma progression to metastatic phenotype. Interestingly, except for I5, cell lines that express high N-cadherin and low E-cadherin levels had a better capacity of proliferation, clonogenicity and migration than cell lines with low N-cadherin and high E-cadherin expression. Thus, consistent with the cadherin switch theory in melanoma progression, our data show a strong link between high N-cadherin/low E-cadherin expression and tumor aggressiveness and defines M2 metastatic melanoma cells (Absent E-cadherin/high N-cadherin expression and highly tumorigenic and motile) as a pertinent model to study the possibility to revert the E- to N-cadherin switch and the metastasis phenotype. In addition to this lymph-node metastasis melanoma model, another cutaneous metastasis melanoma model with low E-cadherin/high N-cadherin expression (i.e. M4T2 cells) was used to further confirm our results.

Treatment of metastatic melanoma cells with PKC inhibitors Gö6976 or Gö6983 resulted in different responses regarding cadherin switch and oncogenic activity of these cells. Gö6976 but not Gö6983 treatment induced the expression of E-cadherin and inhibited the expression of N-cadherin in metastatic melanoma cells. This Gö6976-induced N- to E-cadherin switch was associated with rapid phenotypic and molecular changes that comply with the generally described model of cadherin switch during tumor progression [6, 14–16]. In fact, our results showed that the Gö6976-induced E-cadherin expression and N-cadherin loss were associated with a rapid cell shape modification from an elongated mesenchymal-like structure into a ‘cuboidal’ epithelial-like shape and a strong increase in cell-cell junctions [43–45]. Furthermore, treatment of lymph-node metastatic melanoma cells with Gö6976 induced the translocation of β-catenin from the nucleus to the plasma membrane. However, the membrane β-catenin staining in Gö6976-treated cells is not perfectly lining the plasma membrane. In fact, it has been shown that newly synthesized E-cadherin arrives at the plasma membrane already in complex with β-catenin [46–48]. Then, once at the plasma membrane, this β-catenin pool is transferred to E-cadherins that were already present at the plasma membrane. Furthermore, the β-catenin released from E-cadherin may participate in new exchange cycles [47]. Thus, since during the times of Gö6976-treatment used in our present study (0–48 h), E-cadherin expression is continuing to increase (Fig. 2), so new E-cadherin molecules are still being synthesized and transported to the plasma membrane, the diffuse β-catenin staining may be explained by these β-catenin-E-cadherin exchange cycles [47]. In agreement with this hypothesis, in Gö6976-treated cells, β-catenin staining, which completely disappeared from the nucleus, is mostly accumulated at the newly formed intercellular junctions while less β-catenin staining is observed at the free borders of the plasma membrane or in the cytoplasm, consistent with E-cadherin staining (Fig. 3c). This translocation of the β-catenin from the nucleus to the plasma membrane favors its tumor suppressor role of insuring the stability of E-cadherin-mediated intercellular interactions to its oncogenic role of transcription factor [32, 33], as further supported by the inhibition of the expression of its target cyclin D1 after Gö6976 treatment. These observations also suggest that the Gö6976-induced E-cadherin molecules are functional. In addition to these molecular changes, Gö6976 treatment resulted in decreased anchorage-independent growth and motility of metastatic melanoma cells, confirming the reversion of the metastatic phenotype. In contrast to the metastatic melanoma cells (M2 and M4T2 cell lines), Gö6976 inhibitor did not significantly affect the expression of E- or N-cadherins or the anchorage-independent growth in the primary melanoma cell line (I5) (Additional file 2). Thus, the effects of this inhibitor seem to be specific to advanced metastatic melanoma suggesting that the reversion of the mesenchymal features by Gö6976 requires late event(s) in the melanoma progression. Since a strong increase in the expression of the Gö6976-target « PKD1 » is observed in M2 metastatic cells in comparison to their corresponding primary tumor cells (I5), overexpression of this serine/threonine kinase could be one of these events.

In contrast to Gö6976, the PKC inhibitor Gö6983 did not affect any of the E-/N-cadherin expression, intercellular interactions, β-catenin subcellular localization or anchorage-independent growth in metastatic melanoma cells. Although Gö6983 could significantly inhibit the chemotactic (FBS attraction in transwell) migration of these cells but not their horizontal (wound healing) migration, its effect was much lower than the effect of Gö6976. These results suggest that suppression of specific PKC isoforms inhibited by Gö6976 but not by Gö6983 would be involved in the reversion of the E- to N-cadherin switch and metastatic phenotype in melanoma. Very few studies have attempted to examine the involvement of PKCs in EMT, E/N-cadherin expression regulation and/or cell-cell junctions. To our knowledge, these studies were based on the treatment of the cells with either PKC activator, phorbol ester [28, 49], or PKC inhibitor Gö6976 [29, 50] but not on direct analysis of the role of specific PKC isoforms. In fact, phorbol ester has been shown to induce N-cadherin expression in osteoblasts [28] and EMT in prostate cancer [49]. On the other hand, Gö6976 has been shown to promote formation of cell junctions and inhibit invasion of urinary bladder carcinoma cells [29], suppress S252W FGFR-2 mutation-induced N-cadherin expression in human osteoblasts [50] and inhibit EMT in renal tubular epithelial cells [51]. These studies support our present data and indicate that phorbol ester and Gö6976 targets, i.e. PKCs, can be involved in the E- to N-cadherin switch; however, no specific isoforms could be determined as key players in this process. A recently published study by Jain and Basu 2014 shows, by overexpression or siRNA techniques, that PKCε promotes EMT in breast cancer [52]. However, this isoform can’t be involved in the reversion of the E- to N-cadherin switch observed in our present study because neither Gö6976 nor Gö6983 is a potent inhibitor of PKCε, which implies that, in melanoma cells, other PKC isoform(s) regulate the cadherin switch.

Given the differential effect of the PKC inhibitors Gö6976 and Gö6983, our present data suggest that specific isoenzyme(s) targeted by Gö6976 but not by Gö6983 would be involved in the reversion of the E- to N-cadherin switch and metastatic phenotype in melanoma.

In vitro kinase assays using recombinant full-length PKC isoenzymes α, β, γ, δ, ε, ζ and μ (i.e. PKD1) show that Gö6976 selectively inhibits PKCα (IC_50_ = 2.3 nM), PKCβ (IC_50_ = 6.2 nM) and PKCμ (IC_50_ = 20 nM). However, it does not affect the kinase activity of the PKCγ, δ, ε, and ζ-isozymes even at μM levels [27]. On the other hand, Gö6983 efficiently inhibits several PKC isozymes (IC_50_ = 7 nM for PKCα and PKCβ; 6 nM for PKCγ, 10 nM for PKCδ and 60 nM for PKCζ) but is extremely inefficient in suppressing PKCμ kinase activity in vitro (IC_50_ = 20 μM) [26]. Despite the highly conserved catalytic region of the PKC isoforms, these kinases are differentially targeted by Gö6976 and Gö6983 ATP-competitive inhibitors. That can be due to variability in their regulatory region that might affect the binding of the inhibitor to the kinase domain [53]. Furthermore, although the chemical structures of Gö6976 and Gö6983 are very close, the central aromatic ring is opened in Gö6983 but intact in Gö6976, which accounts for the different substrate specificities of these inhibitors [26]. In contrast to the other PKC isoforms that are not inhibited by Gö6976, conventional PKCs (PKCα and PKCβ) and PKCμ share tandem repetition of C1 motifs (forming the DAG/PMA-binding domain) that is located at a comparable distance towards the catalytic domain [23], which could be important for the binding of Gö6976. On the other hand, PKCμ significantly differs in some structural features from the other PKC isoforms including PKCα and PKCβ [23]. In fact, PKCμ is larger than the other PKCs with a sequence of 912 amino acids. It contains a hydrophobic domain in the N-terminal region and a pleckstrin homology domain that are absent in the other PKC isoforms. This additional sequence may be responsible for the failure of Gö6983 to inhibit PKCμ probably by preventing its binding to the ATP-binding site. Furthermore, the specificity of action of these inhibitors can be independent of the regulatory domain of the PKCs but differences in small sequences or single amino acids in their catalytic domains can affect the affinity of the inhibitors to the ATP-binding site. However, structural/molecular biology studies are required determine the exact reason for the differences in pharmacological action of these inhibitors.

Whatever the mechanism, since Gö6976 is a selective inhibitor of PKCα, PKCβ and PKCμ and Gö6983 a selective inhibitor of PKCα and PKCβ but not PKCμ [26, 27], we hypothesized that the specific inhibition of PKCμ, which is also known as PKD1, would be responsible for the Gö6976-induced N- to E-cadherin switch and tumor reversion in metastatic melanoma cells. Although this serine/threonine kinase is a major cellular target for the tumor-promoting phorbol esters and growth factors which rapidly induce its activity [25, 54], PKD1 has a complex relationship with respect to cancer development. In fact, depending on the tissue type, PKD1 exerts different functions and effects [55]. To date, the role of PKD1 in melanoma initiation and progression is not yet explored, except for the unique data by Kempkes et al. 2012 showing that PKD1 knockdown inhibits proliferation and migration-related processes such as filopodia formation and αvβ3 integrin recycling in WM9 melanoma cell line [56]. These data support our hypothesis. To study more specifically and deeply the involvement of PKD1 in melanoma progression, we firstly analyzed the expression pattern of this kinase in different melanoma cell lines. PKD1 was expressed in 4 out of the 5 melanoma cell lines tested and this expression strongly correlated with E-cadherin negative/N-cadherin positive phenotype and metastatic potential (anchorage-independent growth and migration). PKD1 knockdown in M4T2 metastatic melanoma cells significantly induced down-regulation of N-cadherin and up-regulation of E-cadherin (Additional file 4), supporting the role of PKD1 in E-cadherin to N-cadherin switch. Several studies suggest a role of PKD1 in gene expression regulation either by direct interaction and phosphorylation of transcription factors [57, 58] or by phosphorylation and nuclear exclusion of histone deacetylases (HDACs) [59]. Some of the transcription factors that can be regulated by PKD1 are known to modulate the expression of E-cadherin or N-cadherin even if no direct correlation between PKD1 and these cadherins has been established. In fact, a study by Eiseler et al. 2012 has shown that PKD1 efficiently interacts in nuclei with Snail1, the main transcription factor that suppresses E-cadherin expression during EMT in most epithelial cancers including melanoma [60, 61]. PKD1 phosphorylates Snail1 at Ser-11 which recruits HDAC-1 and −2 as well as LOXL3, a transcriptional co-activator which is also upregulated by PKD1. This newly formed complex enhances Snail activity and induces proliferation and anchorage-independent growth [57]. Although, in this study, authors did not test whether this PKD1-mediated regulation of snail1 activity might affect E-cadherin/N-cadherin expression, another study by Peinado et al. 2004 shows that snail mediates E-cadherin repression by the recruitment of HDAC1/2. Thus, it is very likely that PKD1 might inhibit E-cadherin expression using this mechanism. On the other hand, PKD1 can induce the activation of NFκB [62], a transcription factor that can directly bind to N-cadherin promoter and activate its expression [63]. Interestingly, N-cadherin can also be directly regulated by E-cadherin. In fact, loss of E-cadherin induces NFκB activity and consequent N-cadherin expression in melanoma cells. Thus, regulation of E-cadherin expression by PKD1 could be enough to induce E- to N-cadherin switch. Although a bunch of evidence suggest a role of PKD1 in the regulation of transcription factors that are regulators of E- or N-cadherin expression, these hypotheses need to be tested to determine the exact mechanism by which PKD1 regulates E- to N-cadherin switch.

In the second metastatic melanoma cell line tested (i.e. M2), although the stable knockdown of PKD1 by specific shRNAs induced the expression of E-cadherin, these shPKD1-induced E-cadherin molecules could only be detected as cleaved form in the colony spheres of cells cultured in semi-solid medium or as secreted molecules in the conditioned medium of the adherent cells. Furthermore, the shPKD1-induced E-cadherin molecules did not allow the formation of cell-cell junctions in M2 melanoma cells; on the contrary, the cells transduced by shPKD1 maintained their elongated mesenchymal and isolated shape. Furthermore, although PKD1 knockdown induced a slight decrease in the horizontal migration (wound healing) potential of metastatic melanoma cells, the inhibition was very low in comparison of the effect of the Gö6976 inhibitor. These data suggest that the E-cadherin molecules induced by PKD1 knockdown are not functional, which is not surprising since these molecules were observed in a truncated form. This observation could be explained by the strong expression, by M2 melanoma cells, of metalloproteinases (MMPs) such as MMP2, MMP9, MMP12 and MMP13 [64–67] that may cleave the newly synthesized E-cadherin molecules [68]. Noteworthy, PKCs, in particular PKCα, have been shown to induce MMP expression and/or activity [69–71]. Thus, the restoration of full length and functional E-cadherin molecules by Gö6976 could be the consequence of the dual inhibition of PKD1 (which induces E-cadherin expression) and PKCα (which may inhibit expression and/or activity of MMPs, thus preventing the cleavage of the newly synthesized E-cadherin). The mechanism by which E-cadherin cleavage is mediated in M2 cells might be absent or negatively regulated in M4T2 cells where full-length E-cadherin could be detected after PKD1 knockdown (Additional file 4). Thus, protease profiling and PKC isoform combination silencing experiments would allow the verification of these hypotheses.

Finally, despite its failure to induce the expression of functional E-cadherin, PKD1 knockdown alone strongly altered anchorage-independent growth and migration of metastatic melanoma cells, probably by regulating signaling pathways such as extracellular signal-regulated kinase (ERK), c-jun N-terminal kinase (JNK) and nuclear factor-kappa B (NFκB), as previously described in hormone-positive breast cancer [72, 73] and pancreatic cancer [74, 75].

## Conclusions

The cPKC/PKCμ inhibitor Gö6976 reverses the E- to N-cadherin switch and as a consequence induces profound morphological, molecular and cellular changes that revert the mesenchymal phenotype and metastatic potential of aggressive melanoma cells. On the other hand, the cPKC inhibitor Gö6983 doesn’t show any significant effect on this cadherin switch. The comparison of the target spectrum of these two PKC inhibitors indicates that these observations are not the consequence of the inhibition of cPKCs, but identifies a novel serine/threonine kinase, i.e. PKCμ isoform (also known as PKD1), whose specific inhibition by shRNAs induces E-cadherin expression and reverses the metastatic phenotype in all metastatic melanoma cell lines tested. Noteworthy, in one of these cell lines, specific inhibition of PKD1 alone induces the expression of E-cadherin molecules which are found to be cleaved, probably due to the effect of proteases secreted by these cells. However, the PKD1/cPKC inhibitor Gö6976 induces the expression of full length E-cadherin in this same cell line. Thus, in some melanoma cells, depending on their protease expression profile, a combinatory inhibition of PKD1 and cPKC might be considered for a more effective reversion of the E- to N-cadherin switch.

Despite the recent advances in metastatic melanoma treatments including molecular targeted and immune therapies, many patients develop resistance or do not respond to these therapeutic approaches. Since the E- to N-cadherin switch plays a critical role in the response to these therapies and in melanoma progression and based on our data from the present study, we propose the inhibition of PKD1 as a novel attractive approach for the treatment of metastatic melanoma that should be further tested in pre-clinical studies either alone or in combination with other therapies depending of the disease context.

## Additional files

Additional file 1: Correlation between N-/E-cadherin expression and mesenchymal features in primary (T1 and I5) and metastatic (G1, M2 and M4T2) melanoma cells. The relationship between N-cadherin (A) or E-cadherin (B) expression and mesenchymal features in melanoma cells was estimated using Pearson’s correlation analysis applied on the data from western blot, MTT, methylcellulose and wound healing assays that are presented in Figs. 1, 6 and 7a. In panel B, the last column of the table represents the same correlation study but excluding I5 cell line from the analysis. The results highlight the strong and significant negative correlation between E-cadherin expression and mesenchymal features in most melanoma models and the existence of some models (such as I5) in which E-cadherin loss might not be enough to induce mesenchymal features and might require additional events in tumor development. (PPTX 64 kb)
Additional file 2: Effect of Gö6976 on the expression of E- and N-cadherins and the anchorage-independent growth in I5 primary melanoma cell line. A. I5 primary melanoma cells were seeded in 6-well plates at the density of 50,000 cells per well. After three days of culture, cells were treated with 1 μM Gö6976, 1 μM Gö6983 or DMSO for 24 h. Cells were then lysed and proteins analyzed by western blot using anti-E-cadherin, anti-N-cadherin or anti-actin antibodies. T1 melanoma cells were used as positive control for E-cadherin expression. B. I5 primary melanoma cells were seeded in methylcellulose (1000 cells per plate) containing 1 μM Gö6976 or 1 μM Gö6983. The colonies were counted after 18 days of culture. (PPTX 135 kb)
Additional file 3: Correlation between PKD1 expression and mesenchymal features in primary (T1 and I5) and metastatic (G1, M2 and M4T2) melanoma cells. The relationship between PKD1 expression and mesenchymal features in melanoma cells was estimated using Pearson’s correlation analysis applied on the data from western blot, MTT, methylcellulose and wound healing assays that are presented in Figs. 1, 6 and 7a. (PPTX 51 kb)
Additional file 4: PKD1 knockdown induces the expression of E-cadherin and inhibits the expression of N-cadherin in M4T2 metastatic melanoma cells. M4T2 cells were transfected or not with either specific PKD1-targeting (siPKD1) or control non-targeting (siControl) siRNAs according to the manufacturer protocol (Santa Cruz Biotechnology, sc-36245 and sc-37007, respectively). Three days after transfection, these cells were analyzed by western blot for PKD1, E-cadherin, N-cadherin and actin expression. (PPTX 102 kb)

## Abbreviations

CAMK: Calcium/calmodulin-dependent kinase
DMSO: Dimethylsulfoxide
EMT: Epithelial-mesenchymal transition
ERK: Extracellular signal-regulated kinase
JNK: c-jun N-terminal kinase
MET: Mesenchymal-epithelial transition
MMP: Metalloproteinase
NFκB: Nuclear factor-kappa B
PKC: Protein kinase C
PKD1: Protein kinase D1

## References

[1] Erdei E, Torres SM (2010). A new understanding in the epidemiology of melanoma. Expert Rev Anticancer Ther.

[2] Garbe C, Peris K, Hauschild A, Saiag P, Middleton M, Spatz A (2012). Diagnosis and treatment of melanoma. European consensus-based interdisciplinary guideline--Update 2012. Eur J Cancer.

[3] Gray-Schopfer V, Wellbrock C, Marais R (2007). Melanoma biology and new targeted therapy. Nature.

[4] Callahan MK (2016). Immune checkpoint therapy in melanoma. Cancer J.

[5] Carlino MS, Long GV, Kefford RF, Rizos H (2015). Targeting oncogenic BRAF and aberrant MAPK activation in the treatment of cutaneous melanoma. Crit Rev Oncol Hematol.

[6] Hsu MY, Meier FE, Nesbit M, Hsu JY, Van Belle P, Elder DE (2000). E-cadherin expression in melanoma cells restores keratinocyte-mediated growth control and down-regulates expression of invasion-related adhesion receptors. Am J Pathol.

[7] Valyi-Nagy IT, Hirka G, Jensen PJ, Shih IM, Juhasz I, Herlyn M (1993). Undifferentiated keratinocytes control growth, morphology, and antigen expression of normal melanocytes through cell-cell contact. Lab Invest.

[8] Danen EH, de Vries TJ, Morandini R, Ghanem GG, Ruiter DJ, van Muijen GN (1996). E-cadherin expression in human melanoma. Melanoma Res.

[9] Hsu MY, Wheelock MJ, Johnson KR, Herlyn M (1996). Shifts in cadherin profiles between human normal melanocytes and melanomas. J Investig Dermatol Symp Proc.

[10] Haass NK, Smalley KS, Herlyn M (2004). The role of altered cell-cell communication in melanoma progression. J Mol Histol.

[11] Halbleib JM, Nelson WJ (2006). Cadherins in development: cell adhesion, sorting, and tissue morphogenesis. Genes Dev.

[12] Kemler R (1993). From cadherins to catenins: cytoplasmic protein interactions and regulation of cell adhesion. Trends Genet.

[13] Lee JM, Dedhar S, Kalluri R, Thompson EW (2006). The epithelial-mesenchymal transition: new insights in signaling, development, and disease. J Cell Biol.

[14] Hazan RB, Qiao R, Keren R, Badano I, Suyama K (2004). Cadherin switch in tumor progression. Ann N Y Acad Sci.

[15] Li G, Satyamoorthy K, Herlyn M (2001). N-cadherin-mediated intercellular interactions promote survival and migration of melanoma cells. Cancer Res.

[16] Wheelock MJ, Shintani Y, Maeda M, Fukumoto Y, Johnson KR (2008). Cadherin switching. J Cell Sci.

[17] Alexaki VI, Javelaud D, Van Kempen LC, Mohammad KS, Dennler S, Luciani F (2010). GLI2-mediated melanoma invasion and metastasis. J Natl Cancer Inst.

[18] Miller AJ, Mihm MC (2006). Melanoma. N Engl J Med.

[19] Ciolczyk-Wierzbicka D, Gil D, Laidler P (2012). The inhibition of cell proliferation using silencing of N-cadherin gene by siRNA process in human melanoma cell lines. Curr Med Chem.

[20] Newton AC (1995). Protein kinase C: structure, function, and regulation. J Biol Chem.

[21] Denning MF (2012). Specifying protein kinase C functions in melanoma. Pigment Cell Melanoma Res.

[22] Oka M, Kikkawa U (2005). Protein kinase C in melanoma. Cancer Metastasis Rev.

[23] Johannes FJ, Prestle J, Eis S, Oberhagemann P, Pfizenmaier K (1994). PKCu is a novel, atypical member of the protein kinase C family. J Biol Chem.

[24] Valverde AM, Sinnett-Smith J, Van Lint J, Rozengurt E (1994). Molecular cloning and characterization of protein kinase D: a target for diacylglycerol and phorbol esters with a distinctive catalytic domain. Proc Natl Acad Sci U S A.

[25] Rozengurt E, Rey O, Waldron RT (2005). Protein kinase D signaling. J Biol Chem.

[26] Gschwendt M, Dieterich S, Rennecke J, Kittstein W, Mueller HJ, Johannes FJ (1996). Inhibition of protein kinase C mu by various inhibitors. Differentiation from protein kinase c isoenzymes. FEBS Lett.

[27] Martiny-Baron G, Kazanietz MG, Mischak H, Blumberg PM, Kochs G, Hug H (1993). Selective inhibition of protein kinase C isozymes by the indolocarbazole Go 6976. J Biol Chem.

[28] Delannoy P, Lemonnier J, Hay E, Modrowski D, Marie PJ (2001). Protein kinase C-dependent upregulation of N-cadherin expression by phorbol ester in human calvaria osteoblasts. Exp Cell Res.

[29] Koivunen J, Aaltonen V, Koskela S, Lehenkari P, Laato M, Peltonen J (2004). Protein kinase C alpha/beta inhibitor Go6976 promotes formation of cell junctions and inhibits invasion of urinary bladder carcinoma cells. Cancer Res.

[30] Gassara A, Messai Y, Gaudin C, Abouzahr S, Jalil A, Diarra-Mehrpour M (2006). The decreased susceptibility of metastatic melanoma cells to killing involves an alteration of CTL reactivity. Int J Oncol.

[31] Tian X, Liu Z, Niu B, Zhang J, Tan TK, Lee SR (2011). E-cadherin/beta-catenin complex and the epithelial barrier. J Biomed Biotechnol.

[32] Du W, Liu X, Fan G, Zhao X, Sun Y, Wang T (2014). From cell membrane to the nucleus: an emerging role of E-cadherin in gene transcriptional regulation. J Cell Mol Med.

[33] Orsulic S, Huber O, Aberle H, Arnold S, Kemler R (1999). E-cadherin binding prevents beta-catenin nuclear localization and beta-catenin/LEF-1-mediated transactivation. J Cell Sci.

[34] Sinnberg T, Menzel M, Ewerth D, Sauer B, Schwarz M, Schaller M (2011). beta-Catenin signaling increases during melanoma progression and promotes tumor cell survival and chemoresistance. PLoS One.

[35] Sandig M, Voura EB, Kalnins VI, Siu CH (1997). Role of cadherins in the transendothelial migration of melanoma cells in culture. Cell Motil Cytoskeleton.

[36] Chaffer CL, Thompson EW, Williams ED (2007). Mesenchymal to epithelial transition in development and disease. Cells Tissues Organs.

[37] Hugo H, Ackland ML, Blick T, Lawrence MG, Clements JA, Williams ED (2007). Epithelial--mesenchymal and mesenchymal--epithelial transitions in carcinoma progression. J Cell Physiol.

[38] Thiery JP (2002). Epithelial-mesenchymal transitions in tumour progression. Nat Rev Cancer.

[39] Yao D, Dai C, Peng S (2011). Mechanism of the mesenchymal-epithelial transition and its relationship with metastatic tumor formation. Mol Cancer Res.

[40] Chen A, Beetham H, Black MA, Priya R, Telford BJ, Guest J (2014). E-cadherin loss alters cytoskeletal organization and adhesion in non-malignant breast cells but is insufficient to induce an epithelial-mesenchymal transition. BMC Cancer.

[41] Knudsen KA, Sauer C, Johnson KR, Wheelock MJ (2005). Effect of N-cadherin misexpression by the mammary epithelium in mice. J Cell Biochem.

[42] Hulit J, Suyama K, Chung S, Keren R, Agiostratidou G, Shan W (2007). N-cadherin signaling potentiates mammary tumor metastasis via enhanced extracellular signal-regulated kinase activation. Cancer Res.

[43] Derycke LD, Bracke ME (2004). N-cadherin in the spotlight of cell-cell adhesion, differentiation, embryogenesis, invasion and signalling. Int J Dev Biol.

[44] Islam S, Carey TE, Wolf GT, Wheelock MJ, Johnson KR (1996). Expression of N-cadherin by human squamous carcinoma cells induces a scattered fibroblastic phenotype with disrupted cell-cell adhesion. J Cell Biol.

[45] Wheelock MJ, Johnson KR (2003). Cadherins as modulators of cellular phenotype. Annu Rev Cell Dev Biol.

[46] Hinck L, Nathke IS, Papkoff J, Nelson WJ (1994). Dynamics of cadherin/catenin complex formation: novel protein interactions and pathways of complex assembly. J Cell Biol.

[47] Klingelhofer J, Troyanovsky RB, Laur OY, Troyanovsky S (2003). Exchange of catenins in cadherin-catenin complex. Oncogene.

[48] Ozawa M, Kemler R (1992). Molecular organization of the uvomorulin-catenin complex. J Cell Biol.

[49] He H, Davidson AJ, Wu D, Marshall FF, Chung LW, Zhau HE (2010). Phorbol ester phorbol-12-myristate-13-acetate induces epithelial to mesenchymal transition in human prostate cancer ARCaPE cells. Prostate.

[50] Lemonnier J, Hay E, Delannoy P, Lomri A, Modrowski D, Caverzasio J (2001). Role of N-cadherin and protein kinase C in osteoblast gene activation induced by the S252W fibroblast growth factor receptor 2 mutation in Apert craniosynostosis. J Bone Miner Res.

[51] Tang R, Yang C, Tao JL, You YK, An N, Li SM (2011). Epithelial-mesenchymal transdifferentiation of renal tubular epithelial cells induced by urinary proteins requires the activation of PKC-alpha and betaI isozymes. Cell Biol Int.

[52] Jain K, Basu A (2014). Protein kinase C-epsilon promotes EMT in breast cancer. Breast Cancer.

[53] Steinberg SF (2008). Structural basis of protein kinase C isoform function. Physiol Rev.

[54] Rozengurt E, Sinnett-Smith J, Van Lint J, Valverde AM (1995). Protein kinase D (PKD): a novel target for diacylglycerol and phorbol esters. Mutat Res.

[55] Sundram V, Chauhan SC, Jaggi M (2011). Emerging roles of protein kinase D1 in cancer. Mol Cancer Res.

[56] Kempkes C, Rattenholl A, Buddenkotte J, Strozyk E, Eberle J, Hausser A (2012). Proteinase-activated receptors 1 and 2 regulate invasive behavior of human melanoma cells via activation of protein kinase D1. J Invest Dermatol.

[57] Eiseler T, Kohler C, Nimmagadda SC, Jamali A, Funk N, Joodi G (2012). Protein kinase D1 mediates anchorage-dependent and -independent growth of tumor cells via the zinc finger transcription factor Snail1. J Biol Chem.

[58] Johannessen M, Delghandi MP, Rykx A, Dragset M, Vandenheede JR, Van Lint J (2007). Protein kinase D induces transcription through direct phosphorylation of the cAMP-response element-binding protein. J Biol Chem.

[59] Sinnett-Smith J, Ni Y, Wang J, Ming M, Young SH, Rozengurt E (2014). Protein kinase D1 mediates class IIa histone deacetylase phosphorylation and nuclear extrusion in intestinal epithelial cells: role in mitogenic signaling. Am J Physiol Cell Physiol.

[60] Batlle E, Sancho E, Franci C, Dominguez D, Monfar M, Baulida J (2000). The transcription factor snail is a repressor of E-cadherin gene expression in epithelial tumour cells. Nat Cell Biol.

[61] Poser I, Dominguez D, de Herreros AG, Varnai A, Buettner R, Bosserhoff AK (2001). Loss of E-cadherin expression in melanoma cells involves up-regulation of the transcriptional repressor Snail. J Biol Chem.

[62] Yuan J, Lugea A, Zheng L, Gukovsky I, Edderkaoui M, Rozengurt E (2008). Protein kinase D1 mediates NF-kappaB activation induced by cholecystokinin and cholinergic signaling in pancreatic acinar cells. Am J Physiol Gastrointest Liver Physiol.

[63] Kuphal S, Bosserhoff AK (2006). Influence of the cytoplasmic domain of E-cadherin on endogenous N-cadherin expression in malignant melanoma. Oncogene.

[64] Kondratiev S, Gnepp DR, Yakirevich E, Sabo E, Annino DJ, Rebeiz E (2008). Expression and prognostic role of MMP2, MMP9, MMP13, and MMP14 matrix metalloproteinases in sinonasal and oral malignant melanomas. Hum Pathol.

[65] Rotte A, Martinka M, Li G (2012). MMP2 expression is a prognostic marker for primary melanoma patients. Cell Oncol.

[66] Zhang Z, Zhu S, Yang Y, Ma X, Guo S. Matrix metalloproteinase-12 expression is increased in cutaneous melanoma and associated with tumor aggressiveness. Tumour biology : the journal of the International Society for Oncodevelopmental Biology and Medicine. 2015. doi:10.1007/s13277-015-3622-9 10.1007/s13277-015-3622-9 26040769

[67] Zhao X, Sun B, Li Y, Liu Y, Zhang D, Wang X (2015). Dual effects of collagenase-3 on melanoma: metastasis promotion and disruption of vasculogenic mimicry. Oncotarget.

[68] Hofmann UB, Houben R, Brocker EB, Becker JC (2005). Role of matrix metalloproteinases in melanoma cell invasion. Biochimie.

[69] Kim S, Han J, Lee SK, Choi MY, Kim J, Lee J (2012). Berberine suppresses the TPA-induced MMP-1 and MMP-9 expressions through the inhibition of PKC-alpha in breast cancer cells. J Surg Res.

[70] Roy S, Chakraborti T, Chowdhury A, Chakraborti S (2013). Role of PKC-alpha in NF-kappaB-MT1-MMP-mediated activation of proMMP-2 by TNF-alpha in pulmonary artery smooth muscle cells. J Biochem.

[71] Shin Y, Yoon SH, Choe EY, Cho SH, Woo CH, Rho JY (2007). PMA-induced up-regulation of MMP-9 is regulated by a PKCalpha-NF-kappaB cascade in human lung epithelial cells. Exp Mol Med.

[72] Karam M, Bieche I, Legay C, Vacher S, Auclair C, Ricort JM (2014). Protein kinase D1 regulates ERalpha-positive breast cancer cell growth response to 17beta-estradiol and contributes to poor prognosis in patients. J Cell Mol Med.

[73] Karam M, Legay C, Auclair C, Ricort JM (2012). Protein kinase D1 stimulates proliferation and enhances tumorigenesis of MCF-7 human breast cancer cells through a MEK/ERK-dependent signaling pathway. Exp Cell Res.

[74] Guha S, Tanasanvimon S, Sinnett-Smith J, Rozengurt E (2010). Role of protein kinase D signaling in pancreatic cancer. Biochem Pharmacol.

[75] Kisfalvi K, Hurd C, Guha S, Rozengurt E (2010). Induced overexpression of protein kinase D1 stimulates mitogenic signaling in human pancreatic carcinoma PANC-1 cells. J Cell Physiol.

