# Supplementary material for: Protein kinase C inhibitor Gö6976 but not Gö6983 induces the reversion of E- to N-cadherin switch and metastatic phenotype in melanoma: identification of the role of protein kinase D1
Source: BMC Cancer. 2017 Jan 5;17:12. doi: 10.1186/s12885-016-3007-5 (PMC5217271; doi:10.1186/s12885-016-3007-5)
Supplement: Additional file 1: — Correlation between N-/E-cadherin expression and mesenchymal features in primary (T1 and I5) and metastatic (G1, M2 and M4T2) melanoma cells. The relationship between N-cadherin (A) or E-cadherin (B) expression and mesenchymal features in melanoma cells was estimated using Pearson’s correlation analysis applied on the data from western blot, MTT, methylcellulose and wound healing assays that are presented in Figs. 1, 6 and 7a. In panel B, the last column of the table represents the same correlation study but excluding I5 cell line from the analysis. The results highlight the strong and significant negative correlation between E-cadherin expression and mesenchymal features in most melanoma models and the existence of some models (such as I5) in which E-cadherin loss might not be enough to induce mesenchymal features and might require additional events in tumor development. (PPTX 64 kb) [file 12885_2016_3007_MOESM1_ESM.pptx]

## Slide 1
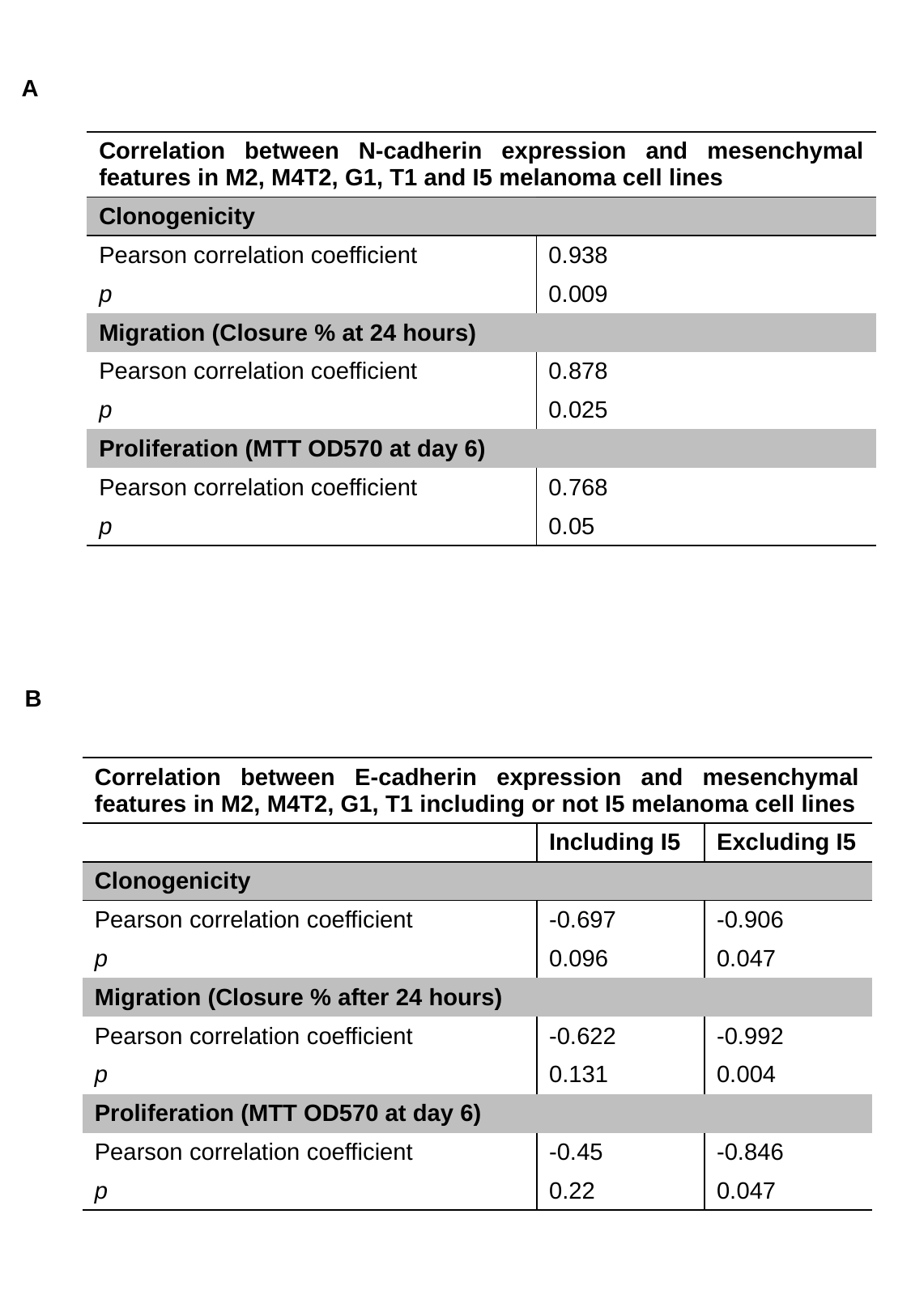

A
| Correlation between N-cadherin expression and mesenchymal features in M2, M4T2, G1, T1 and I5 melanoma cell lines | |
| --- | --- |
| Clonogenicity | |
| Pearson correlation coefficient | 0.938 |
| p | 0.009 |
| Migration (Closure % at 24 hours) | |
| Pearson correlation coefficient | 0.878 |
| p | 0.025 |
| Proliferation (MTT OD570 at day 6) | |
| Pearson correlation coefficient | 0.768 |
| p | 0.05 |
B
| Correlation between E-cadherin expression and mesenchymal features in M2, M4T2, G1, T1 including or not I5 melanoma cell lines | | |
| --- | --- | --- |
| | Including I5 | Excluding I5 |
| Clonogenicity | | |
| Pearson correlation coefficient | -0.697 | -0.906 |
| p | 0.096 | 0.047 |
| Migration (Closure % after 24 hours) | | |
| Pearson correlation coefficient | -0.622 | -0.992 |
| p | 0.131 | 0.004 |
| Proliferation (MTT OD570 at day 6) | | |
| Pearson correlation coefficient | -0.45 | -0.846 |
| p | 0.22 | 0.047 |
