# Supplementary figures and images for: Protein kinase C inhibitor Gö6976 but not Gö6983 induces the reversion of E- to N-cadherin switch and metastatic phenotype in melanoma: identification of the role of protein kinase D1
Source: BMC Cancer. 2017 Jan 5;17:12. doi: 10.1186/s12885-016-3007-5 (PMC5217271; doi:10.1186/s12885-016-3007-5)

## Slide 1
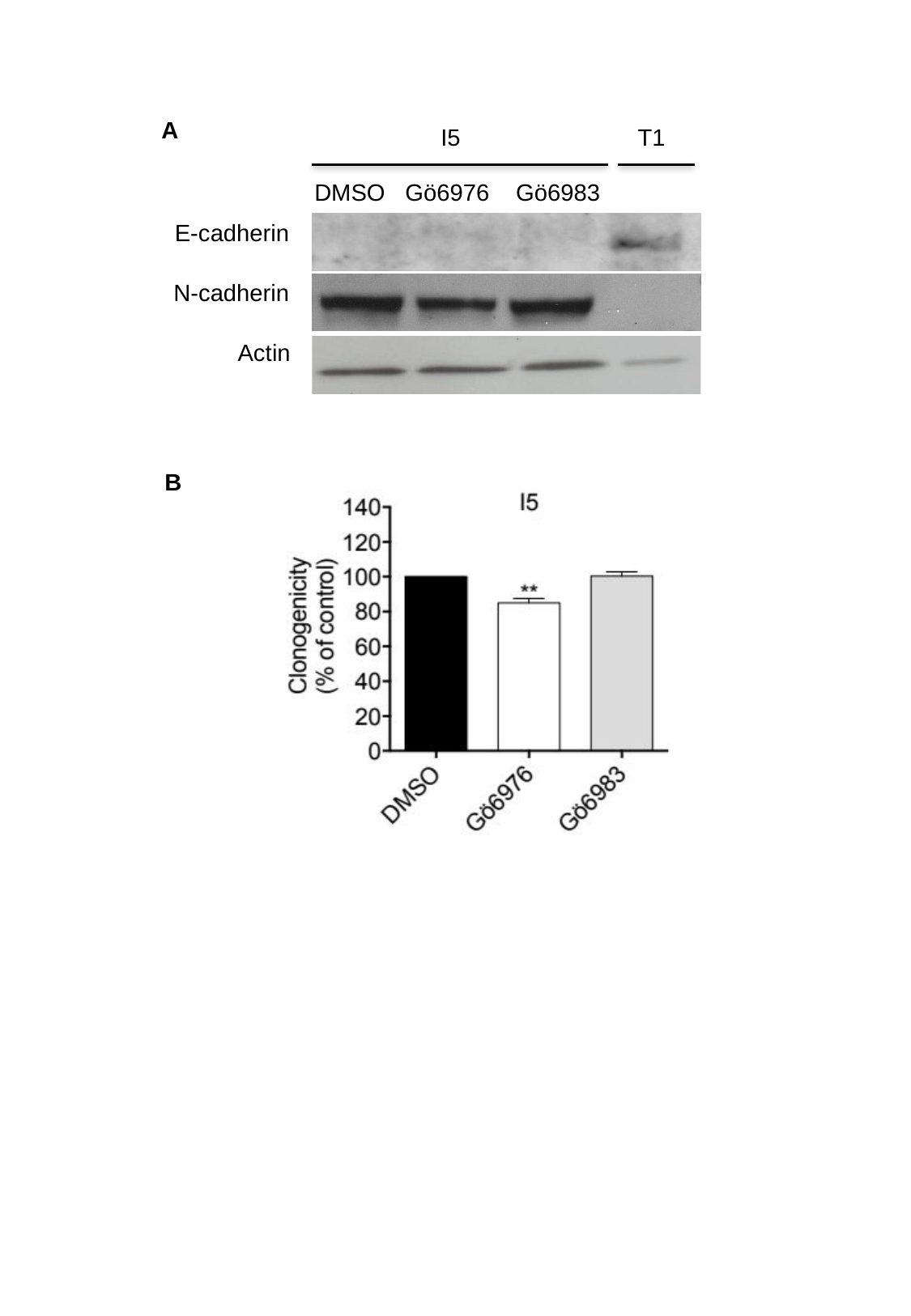

A
T1
I5
DMSO Gö6976 Gö6983
E-cadherin
N-cadherin
Actin
B

Supplement: Additional file 2: — Effect of Gö6976 on the expression of E- and N-cadherins and the anchorage-independent growth in I5 primary melanoma cell line. A. I5 primary melanoma cells were seeded in 6-well plates at the density of 50,000 cells per well. After three days of culture, cells were treated with 1 μM Gö6976, 1 μM Gö6983 or DMSO for 24 h. Cells were then lysed and proteins analyzed by western blot using anti-E-cadherin, anti-N-cadherin or anti-actin antibodies. T1 melanoma cells were used as positive control for E-cadherin expression. B. I5 primary melanoma cells were seeded in methylcellulose (1000 cells per plate) containing 1 μM Gö6976 or 1 μM Gö6983. The colonies were counted after 18 days of culture. (PPTX 135 kb) [file 12885_2016_3007_MOESM2_ESM.pptx]

## Slide 1
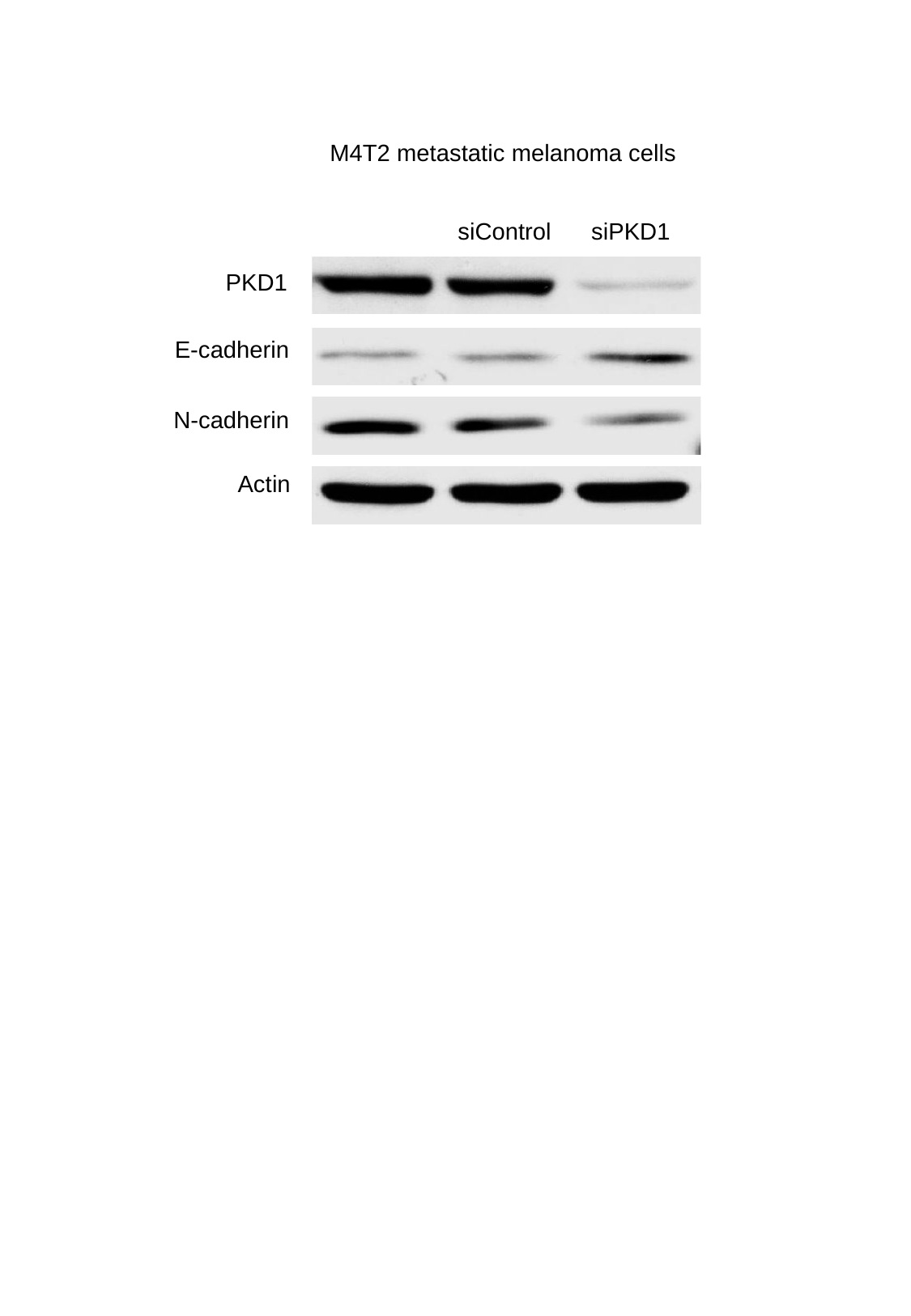

M4T2 metastatic melanoma cells
siControl siPKD1
PKD1
E-cadherin
N-cadherin
Actin

Supplement: Additional file 4: — PKD1 knockdown induces the expression of E-cadherin and inhibits the expression of N-cadherin in M4T2 metastatic melanoma cells. M4T2 cells were transfected or not with either specific PKD1-targeting (siPKD1) or control non-targeting (siControl) siRNAs according to the manufacturer protocol (Santa Cruz Biotechnology, sc-36245 and sc-37007, respectively). Three days after transfection, these cells were analyzed by western blot for PKD1, E-cadherin, N-cadherin and actin expression. (PPTX 102 kb) [file 12885_2016_3007_MOESM4_ESM.pptx]
