# Supplementary material for: Protein kinase C inhibitor Gö6976 but not Gö6983 induces the reversion of E- to N-cadherin switch and metastatic phenotype in melanoma: identification of the role of protein kinase D1
Source: BMC Cancer. 2017 Jan 5;17:12. doi: 10.1186/s12885-016-3007-5 (PMC5217271; doi:10.1186/s12885-016-3007-5)
Supplement: Additional file 3: — Correlation between PKD1 expression and mesenchymal features in primary (T1 and I5) and metastatic (G1, M2 and M4T2) melanoma cells. The relationship between PKD1 expression and mesenchymal features in melanoma cells was estimated using Pearson’s correlation analysis applied on the data from western blot, MTT, methylcellulose and wound healing assays that are presented in Figs. 1, 6 and 7a. (PPTX 51 kb) [file 12885_2016_3007_MOESM3_ESM.pptx]

## Slide 1
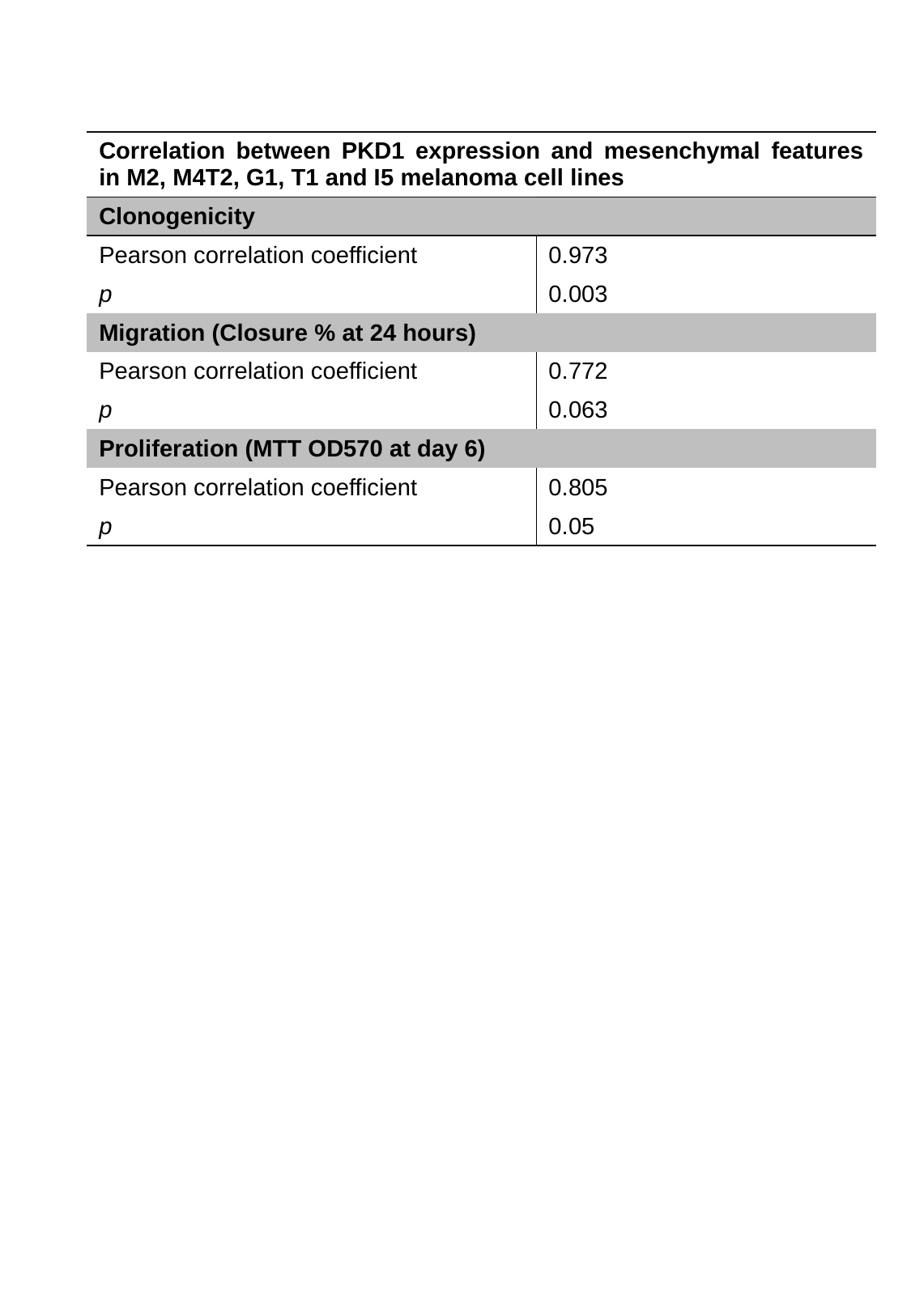

| Correlation between PKD1 expression and mesenchymal features in M2, M4T2, G1, T1 and I5 melanoma cell lines | |
| --- | --- |
| Clonogenicity | |
| Pearson correlation coefficient | 0.973 |
| p | 0.003 |
| Migration (Closure % at 24 hours) | |
| Pearson correlation coefficient | 0.772 |
| p | 0.063 |
| Proliferation (MTT OD570 at day 6) | |
| Pearson correlation coefficient | 0.805 |
| p | 0.05 |
